# Engineered calcium carbonate-modiated SAzymes with Mn-based single-atom sites for ultrasound-enhanced nanocatalytic therapy

**DOI:** 10.1016/j.mtbio.2025.102463

**Published:** 2025-10-30

**Authors:** Xiao Wang, Bingyu Xu, Fang Li, Zhao Wang, Quanxiang Han, Yuxiao Li, Yen Leng Pak, Yurong Guo, Yingying Jing, Xing Gao, Lei Yu, Jibin Song

**Affiliations:** a School of Biological and Chemical Engineering, Qilu Institute of Technology, Jinan, 250200, PR China; b College of Chemical Engineering and Materials, Shandong University of Aeronautics, Binzhou, 256603, PR China; c State Key Laboratory of Chemical Resource Engineering, College of Chemistry, Beijing University of Chemical Technology, Beijing, 10010, PR China

**Keywords:** Mn-based single-atom sites, Peroxidase-like activity, Cavitation effect, pH-responsive property, Calcium-mediated cell death

## Abstract

The construction of highly effective therapeutic nano-systems will be of great interest in the investigation of cancer treatment; however, developing such the platforms face significant challenges originated from the strict tumor microenvironment (TME). In this study, the engineered carbonate-mediated SAzyme based on UiO-66-NH_2_ with Mn-based single-atom sites and calcium carbonate (CaCO_3_@Mn-UiO-66-NH_2_, CUM) is successfully developed for dual imaging-guided ultrasound-enhanced nano-catalytic therapy. The designed SAzyme with uniform structure and morphology exhibit good stability and suitable particles sizes. The Mn based single-atoms coordinated in the CUM show excellent peroxidase (POD)-like activity to generate high toxic hydroxyl radical (∙OH) in the TME, and its reactive oxygen species (ROS)-based therapeutic effect can be promoted by the cavitation effect generated from ultrasound (US) irradiation. Owing to the pH-responsive property of CUM in the acidic TME, the coated calcium carbonate-based shell is degraded and then released high concentration of Ca^2+^ ions to induce calcium-mediated cell death. The *in vitro* and *in vivo* experiments also confirm a significant increase in apoptosis following CUM intervention, thereby further enhancing the anti-tumor effect upon exposure to US irradiation. The genomic analysis of cancer cells following CUM treatment further identified key genes and associated pathways, which utilize the approach to explore the therapeutic mechanism of the SAzyme. Combined with the synergistic anti-tumor performance of US-activated CUM, this SAzyme provides potential to more effectively inhibit both the initiation and progression of cancer.

## Highlights

• The carbonate-modiated SAzyme (CUM) is constructed through the solvothermal and vapor diffusion methods.
• With US irradiation, CUM showed enhanced POD-like activity for antitumor performance because of the cavitation effect.
• The pH-responsive Ca^2+^ release of CUM in acidic TME can induce calcium-mediated cell death to achieve synergistic therapy.

## Graphical abstract

**Figure:**
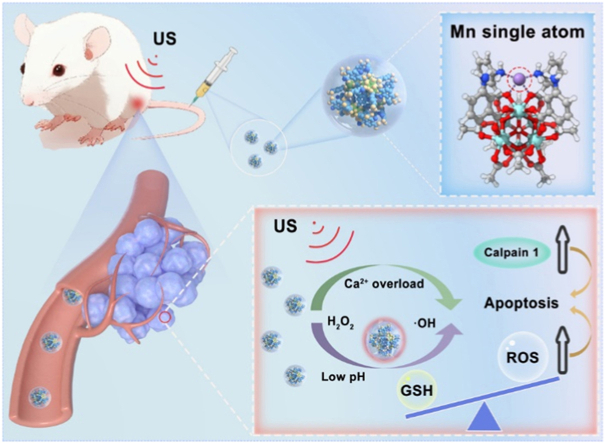

## 1 Introduction

Tumors represent the second leading cause of mortality worldwide and pose a significant threat to global public health. According to projections by the World Health Organization, it is estimated that 13.1 million individuals will die from cancer annually by 2030. Traditional cancer therapy like surgery, chemotherapy, or radiotherapy remains the predominant strategy for inhibiting cancer progression; however, its efficacy is limited by suboptimal therapeutic outcomes and considerable adverse effects. Therefore, the development of novel and effective therapeutic approaches for anti-tumor treatment is of critical importance [[1], [2], [3]]. In recent years, various therapeutic modalities such as photothermal therapy (PTT) [4], photodynamic therapy (PDT) [5], radiotherapy (RT), and sonodynamic therapy (SDT) have been increasingly applied in cancer treatment. Despite notable advancements in these therapeutic strategies, achieving effective outcomes remains challenging due to the complex and dynamic nature of TME [6]. Unlike normal tissues, the TME exhibits distinct biophysical and biochemical characteristics that promote cancer cell proliferation, including elevated levels of hydrogen peroxide (H_2_O_2_), hypoxia, and acidosis [[7], [8], [9]]. Consequently, there is a pressing need to develop biocompatible, TME-responsive nanomaterial-based therapeutic agents for more effective disease intervention.

As evidenced by an increasing body of research, enzyme-based therapies have emerged as particularly promising candidates for addressing a wide range of pathologies. However, naturally occurring enzymes are inherently limited by their susceptibility to environmental factors such as temperature, pH, and ionic strength. Moreover, they face significant challenges including immunogenic reactions and proteolytic degradation [10,11]. In contrast, nanozymes offer several advantages over natural enzymes, including lower production costs, greater stability, scalability in manufacturing, and multifunctional capabilities, making them strong contenders for replacing natural enzymes in therapeutic applications [12]. Nanozymes, such as manganese oxide (MnO_2_), cerium oxide (CeO_2_), cobalt oxide (Co_3_O_4_), and ferric oxide (Fe_3_O_4_), typically exhibit various enzyme-like activities, including peroxidase (POD), catalase (CAT), and oxidase (OD) activity. These properties enable them to interact with the tumor microenvironment (TME), thereby enhancing their potential for application in cancer therapy [8]. Among them, single-atom nanozymes (SAzymes) have emerged as promising candidates for tumor catalytic therapy [13,14]. The exceptional catalytic performance of Mn-based nanozymes originates from the unique multivalent redox chemistry and high-spin electronic configuration of manganese characteristics that also account for its essential role as a cofactor in numerous metalloenzymes, thus establishing these nanoscale catalysts as leading candidates in oncological therapeutic strategies [[15], [16], [17]]. Moreover, the integration of nanozymes within hierarchically porous metal-organic frameworks (MOFs), as demonstrated by Yu et al. through the innovative design of MOF-nanozyme composites exhibiting remarkable catalytic activity and operational stability, represents a significant advancement in the development of advanced therapeutic platforms with enhanced functional capabilities [[18], [19], [20], [21], [22]]. Additionally, due to their specific responsiveness to the tumor microenvironment (TME), various therapeutic modalities such as chemodynamic, photodynamic, photothermal, and sonodynamic therapies have shown promising tumor-selective effects [23]. Nevertheless, the design of nanozymes with high catalytic efficiency remains crucial for improving anti-tumor efficacy.

Although nanozymes possess inherent catalytic advantages that offer considerable therapeutic potential, their use as standalone agents is limited. The key challenge lies in achieving tumor-specific delivery, which critically determines both therapeutic efficacy and systemic safety. Addressing this requires the development of advanced nano-engineering calcium-mediated strategies to enable precise biodistribution. Current calcium-mediated nanomaterials, mainly based on calcium carbonate (CaCO_3_), calcium peroxide (CaO_2_), or calcium phosphate (CaP), exhibit pH-responsive dissolution in the acidic tumor microenvironment (TME), thereby achieving targeted cancer therapy [24]. At the same time, excessive physiological concentrations of Ca^2+^ ions are released, triggering pathological calcium overload to subsequently initiate cascades of calcium-related pathologies. During cellular stress responses, mitochondrial calcium overload induces the collapse of mitochondrial membrane potential (MMP) via the opening of permeability transition pores, simultaneously promoting bursts of reactive oxygen species (ROS) production that contribute to increased oxidative damage [25,26]. In addition, the elevated intramitochondrial Ca^2+^ concentrations can activate calcium/calmodulin-dependent protein kinase II (CaMKII), which subsequently phosphorylates dynamin-related protein 1 (Drp1) at the serine 616 residue. This modification enhances mitochondrial fission, a process that plays a critical role in the activation of apoptotic signaling pathways [27].

**Scheme 1:**
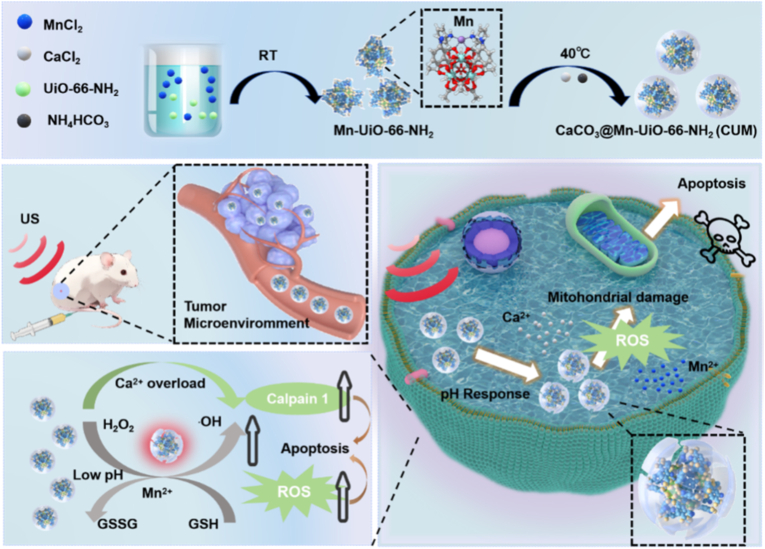
Schematic illustration of the preparation processes of CaCO_3_@Mn-UiO-66-NH_2_**(**CUM), and the underlying tumor killing mechanism of CUM via the combination therapy.

Building upon these advancements, this study presents a responsive “switch-on” therapeutic nanocomposite platform specifically engineered for tumor-targeted therapy. By integrating pH-responsive calcium release with catalytic nanozyme functionalities, the system enables precise spatiotemporal control over therapeutic activation. Herein, an Mn-containing single-atom SAzyme-based on the UiO-66-NH_2_ framework is successfully constructed with an outer CaCO_3_ coating or shell, which serves as a pH-responsive gate. In the weakly acidic TME, this SAzyme can catalyse the conversion of intracellular H_2_O_2_ into ∙OH through a POD-like activity, which can generate ROS and effectively kill tumor cells. Furthermore, this damage to the tumor cells is exacerbated by intracellular calcium overload. As shown in Scheme 1, under this multi-modal management, the tumor cell-killing effect is further enhanced.

## 2 Experimental section

### 2.1 Reagents and materials

CaCl_2_ and NH_4_HCO_3_ are provided by Sinopharm Chemical Reagent Co., Ltd. (China). Manganese chloride (MnCl_2_) is provided by J and K Chemical Co. FDA/propidium iodide (PI) was purchased from SolarBio. MEM and DMEN medium are purchased from Thermo Fisher Scientific, Inc. Fluo-4 AM is purchased from KeyGen Biotech Co., Ltd. 5,5′,6,6′-Tetrachloro-1,1′,3,3′-tetraethylbenzimidazolyl-carbocyanine chloride (JC-1) is purchased from Solarbio.

### 2.2 Cell lines and animals

The Mouse lung fibroblast cells (L929 cells) and 4T1 mouse breast cancer cells (4T1 cells) used in the experiments are sourced from the Cell Bank of the Chinese Academy of Sciences. The experimental mice are female BALB/c mice purchased from Jinan Jinfeng Experimental Animal Co., LTD. All *in vivo* experiments comply with the regulations stipulated in “The National Regulation of China for the Care and Use of Laboratory Animals”.

### 2.3 Synthesis

#### 2.3.1 Synthesis of CUM

UiO-66-NH_2_ is synthesized by the original laboratory formulation. 2.5 g of manganese chloride (MnCl_2_) and 0.05 g of UiO-66-NH_2_ are accurately weighed and dissolved in absolute ethanol. Set the rotation speed to 200–300 revolutions per minute (rpm) and ensure the reaction time is at least 8 h to guarantee sufficient reaction at room temperature. After the reaction, centrifugation is performed several times to remove unbound Mn^2+^.

50 mg Mn-UiO-66-NH_2_ and 150 mg anhydrous calcium chloride are weighed and dissolved in absolute ethanol, sonicated to make the samples completely dissolved and stirred at 300 rpm over 12 h. The products are subsequently collected and centrifuged at 9000 rpm for 5 min to remove the supernatant, and the precipitate is washed with absolute ethanol twice to remove unbound calcium ions. Then it is dissolved again in 50 mL of absolute ethanol, and then 2.5 g of ammonium bicarbonate is put into a 15 mL beaker, and the above two beakers are placed together in a closed environment for 39–40 °C reaction for 24 h. After the reaction, the supernatant is centrifuged, removed, and the precipitate is washed with absolute ethanol several times and deionized water several times. Finally, the product is lyophilized in a vacuum freeze dryer for 12 h to obtain the product CUM.

### 2.4 Characterisation

The morphology and size of the CUM are analyzed using Field emission scanning electron microscope Sigma 300 (Carl Zeiss, Germany) and a JEM-1400 transmission electron microscope (TEM, JEOL, Japan). The size of the CUM is analyzed using Image J. X-ray photoelectron spectroscopy (XPS) spectra are obtained using a Thermo Fisher Scientific ESCALAB 250Xi XPS system. The concentration of Ca^2+^ in the CUM is determined using inductively coupled plasma mass spectrometry (ICP-MS, Jena, PlasmaQuant® MS). Cell uptake is measured using a confocal laser-scanning microscope (CLSM ZEISS LSM 780, Carl Zeiss, Jena, Germany).

### 2.5 Release performance of Ca^2+^

CUM (1 mg) are immersed in PBS (10 mL) at 37 °C with different pH (7.4, 6.5, 5.0) value. The amount of released Ca^2+^ is measured by Inductively Coupled Plasma Mass Spectrometry (ICP-MS), which are collected from the supernatants at 0, 4, 12, and 24 h. The release fractions of Ca^2+^ are calculated as the amount of released Ca^2+^ from CUM divided by the total amount of Ca^2+^ in CUM.

### 2.6 POD-mimic activity of CUM and kinetic assay

The POD-mimic activity of CUM are performed by testing the absorbance at 650 nm containing various concentrations of CUM (50–1000 μg/mL), H_2_O_2_, and TMB under different US irradiation times, which utilize TMB as the ROS indicator. The absorbance of productions at 650 nm is monitored at different processing methods, and the catalytic parameters are calculated by fitting the absorbance data to the Michaelis-Menten equation.

### 2.7 GSHO_x_-mimic activity of CUM

CUM is mixed with GSH (1 mM) in 2 mL of PBS solution to explore the GSHO_x_-mimic activity. DTNB (100 μg/mL) is utilized as an indicator for the GSH consumption. At different CUM concentrations (0, 20, 40, 80, 120, 160, 200 μg/mL), the absorbance values are recorded through UV–Vis–NIR spectrometer.

CUM (1 mg/mL) and GSH (100 mg) were reacted in a 40 mL reaction system for different time periods (0, 4, 12, 24 h). After the reaction time ended, the supernatant of the reaction solution was taken to react with DTNB (100 μg/mL), and the color change was observed and recorded. The absorbance values were recorded through UV–Vis–NIR spectrometer.

### 2.8 Cell culture

L929 cells are cultured in MEM medium containing 10 % fetal bovine serum, while 4T1 cells are incubated in DMEM medium containing 10 % fetal bovine serum. Then, both cell lines were maintained in a humidified incubator with 5 % CO_2_ at 37 °C for subsequent *in vitro* experiments.

### 2.9 Cytotoxicity of CUM

The 4T1 cell suspension is seeded at 1000–10000 cells per well into a 96-well plate with a volume of 200 μL per well. Then, the different concentrations of CUM are added into the 96-well plates in 37 °C, 5 %CO_2_ incubator for 24 h. Add 10 μL of 3-(4,5-dimethyl-2-thiazolyl)-2,5-diphenyltetrazolium bromide (MTT) solution (5 mg/mL prepared in PBS, pH = 7.4), continued incubation for 4 h, carefully discard the well, add 100 μL DMSO per well, and shake for 10 min to fully melt the crystals. Select 490 nm wavelength, measure the light absorption value of each well on the enzyme-linked immunomonitor, record the results, and draw the cell growth curve with the concentration as the abscissa and the light absorption value as the ordinate.

### 2.10 Cellular uptake of CUM

4T1 cells are cultured with for PBS, US, UM, CUM, and CUM + US. Subsequently, Fluo-4 AM (1 μM) was used to label cells after incubation for 4 h. Finally, fluorescence images are visualized by CLSM after co-incubation. In addition, the Image J software is used for the fluorescence intensity analysis.

### 2.11 Detection of ROS production

Various intracellular ROS generation conditions (PBS, US, UM, CUM, and CUM + US) are detected using dichloro-dihydro-fluorescein diacetate (DCFH-DA). All the concentration of experimental samples are 200 μg/mL, and the US power is set as 1.5 W/cm^2^ (US: 1.0 MHz, 20 % duty cycle). The 4T1 cells plated on confocal dishes are co-incubated with CUM for 12 h in the dark. At last, the cell images are obtained from CLSM.

### 2.12 Evaluation of mitochondrial function

The incubated 4T1 cells are divided into four groups (PBS, US, UM, CUM, and CUM + US), and then the prepared JC-1 solutions (10 μg/mL) are added into the cells, respectively. After 10 min of co-incubation time, the mitochondrial membrane potentials of different groups are visualized using CLSM images.

### 2.13 Live/dead cell staining assay

4T1 cells are seeded in 96-well plates at a density of 1 × 10^4^ cells/well and cultured for 24 h. After two washes with PBS buffer, a series of functional nanoparticles are added for an additional 4 h. The nanoparticles that are not taken up by the cells can be washed away and the new culture mediums are re-added. Following the intervention in the ultrasound radiation treatment group, the culture is maintained in a 37 °C, 5 % CO_2_ incubator for 20 h. After being washed carefully twice with PBS buffer, 50 μL of PBS buffer containing FDA (8 μg/mL, which stains live cells green) and PI (20 μg/mL, which stains dead cells red) was added to the sample for staining over a period of 5 min. The staining solution is carefully removed, and the sample is subsequently washed twice with PBS buffer. Following this, fresh PBS buffer is added, and the sample is examined under a fluorescence microscope.

### 2.14 Cell apoptosis assay

Cell apoptosis is assessed using an Annexin V-FITC/PI apoptosis detection kit. 4T1 cells are seeded in 6-well plates at a defined initial density and incubated for 24 h. Subsequently, the culture medium is replaced with DMEM containing 200 μg/mL of PBS, US, UM or CUM/CUM + US. After incubation for 4 h, these cells are divided into sonication group (1.5 W/cm^2^, 3 min) and no sonication group. After another cultivation for 20 h, cells are harvested and followed with the protocol of Annexin V-FITC/PI apoptosis kit for cell apoptosis detection by flow cytometric.

### 2.15 Western blot analysis

The total protein is extracted from transfected cells using radioimmunoprecipitation assay lysis buffer (Beyotime, China). The blots are incubated with primary antibodies at 4 °C. The following antibody diluents are used: anti-Bax (1:1000; cat.no.2772T; Cell Signaling Technology), anti-Bcl-2 (1:1000; cat.no.3498T; Cell Signaling Technology), anti-β-actin (1:1000; cat.no. 4967S; Cell Signaling Technology), anti-Calpain 1 (1:2000; cat.no.BS40546; Bioworld Technology, Inc, USA), anti-TNF-α(1:1000; cat.no.BS1857; Bioworld Technology, Inc, USA) and anti-p53 (1:1000; cat.no.MB9401; Bioworld Technology, Inc, USA). Following incubation for 2 h with goat anti-rabbit IgG H&L horseradish peroxidase conjugate secondary antibody (1:6000; cat. no. BS13278; Bioworld Technology, Inc, USA), the protein bands are visualized using BeyoECL Plus (Beyotime Institute of Biotechnology, China).

### 2.16 NIR-II fluorescence imaging

Excessive ICG and CUM (1 mg/mL) were reacted under room temperature and light protection, with a speed of 300 rpm for more than 6 h. The two were combined by physical adsorption, and then dialysis was performed to remove the unbound ICG. To investigate the distribution and degradation of CUM *in vivo*,100 μL of the solutions of CUM + ICG (200 μg/mL) was tail vein injection into the 4T1 tumor-bearing mice (n = 5), and their near-infrared II (NIR-II) images are performed at 0, 12, 24, and 48 h of post-injection.

### 2.17 MR-imaging

The MRI performance of CUM *in vitro* and *in vivo* was tested by 9.4 *T* magnetic resonance imaging system (BioSpec 94/30 USR). At first, 0.5 mL of H_2_O_2_ (50 μM) was added into 0.5 mL of CUM solution (*C*_Mn_ = 0, 0.04, 0.08, 0.12, 0.2 mM). Then, the mixture was reacted for 10 min and detected by 9.4 *T* magnetic resonance imaging system.

A tumor-bearing mouse model is established by injecting 4T1 cells into the right hind limb of female nude mice at a density of 1 × 10^6^ cells per 200 μL. Once the tumor volume reaches over 150 mm^3^, varying concentrations of CUM (200 μg/mL) are administered to the tumor-bearing mice under anesthesia. And fitted curve plotting was performed by combining relaxation time with varying Mn content. Moreover, the MRI images of CUM *in vivo* were observed by 9.4 *T* magnetic resonance imaging system.

### 2.18 In vivo tumor inhibition

A total of 20 female BALB/C mice weighing approximately 18 g are purchased from Jinfeng Experimental Animal Co., LTD located in Jinan, and these mice are used as the tumor-bearing models for subsequent experiments *in vivo*. Then, 150 μL of 4T1 cells (1 × 10^6^) are implanted into the right hips of these mice. Seven days of post-inoculation, 4T1 tumor-bearing mice were subjected to the following treatments: PBS, US, UM, CUM, and CUM with US irradiation. The concentrations of used nanomaterials are set at 200 μg/mL, 100 μL (1 mg/kg). The body weights and tumor sizes of each mouse are measured once every two days with a scale and caliper. After 21 days, the mice are euthanized by injection of sodium pentobarbital (150–200 mg/kg). Subsequently, the tumor tissues from the experimental mice are excised, documented through photography, and weighed. The tumor volumes of each group are calculated as volume = (tumor width)^2^ × (tumor length)/2.

### 2.19 H&E and TUNEL staining

The tumor tissues extracted from the mice of different groups are fixed in 4 % paraformaldehyde solution for 48 h, embedded in paraffin, and sections. And then, these sections are dewaxed and rehydrated. Subsequently, the cell nuclei were counterstained with hematoxylin for 10 min, followed by cytoplasmic staining with eosin for 5 min. At the same time, PBS, US, UM, CUM and CUM + US groups were separated for TUNEL staining. Finally, the slides images are examined and captured by the Leica TCS SP8.

### 2.20 Biochemical analysis of blood

Fresh blood samples are obtained from the mice of CUM group after *i.v.* injection time (0, 15, and 30 days) by the blood collection via ocular puncture, and they are analyzed by the biochemical analysis to confirm the biosafe of CUM.

### 2.21 Statistical analysis

Data are presented as mean ± standard deviation. Statistical significance between the control and experimental groups was assessed using Student's t-test for single-column analyses and two-way ANOVA for grouped analyses, performed with GraphPad Prism 8.0 software. (∗*P* < 0.05, ∗∗*P* < 0.01, and ∗∗∗∗*P* < 0.0001).

## 3 Results and discussion

### 3.1 Preparation and characterisation of CUM

**Fig. 1:**
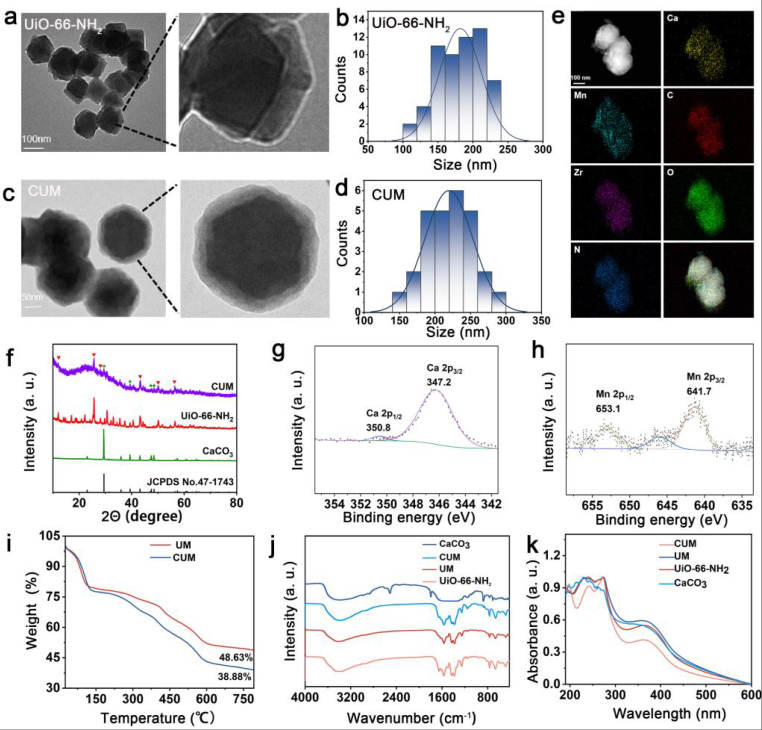
Synthesis and characterisation of CUM. (a) TEM images and (b) particle size analysis of UiO-66-NH_2_. (c) TEM images and (d)particle size analysis of CUM. (e) EDS mapping analysis of CUM. (f) XRD patterns of UiO-66-NH_2,_ CaCO_3_ and CUM. XPS spectra. (g) Ca 2p and (h) Mn 2p of CUM. (i) Thermogravimetric analysis of UM and CUM. (j) FT-IR spectra and (k) UV spectroscopy of the UiO-66-NH_2_, CaCO_3_, UM and CUM.

Due to its biocompatibility and selective responsiveness to the acidic TME, Ca^2+^ overload-mediated cancer treatment provides sufficient advantages [28,29]. Therefore, CaCO_3_ is selected as the shell material for the synthesis of nanomaterials, as illustrated in Scheme 1, which presents the detailed preparation process of CaCO_3_@Mn-UiO-66-NH_2_ (CUM). In comparison with other series of MOFs, UiO-66-NH_2_ are well-defined microporous materials with a controlled size that are widely used as drug-carriers. The synthesis of UiO-66-NH_2_ are according to the previous articles [30], which SEM and TEM images revealed their uniform structure with the diameter of 190 nm (Fig. 1a and b). After the solvothermal reaction of Mn^2+^ ions and UiO-66-NH_2_, Mn-UiO-66-NH_2_ (UM) containing the Mn single-atom sites can be obtained, in which the ions are bonded with the animo groups of UiO-66-NH_2_ through the formation of coordination bonds. Then, the preparation of CUM is subsequently carried out through the gas diffusion method, which facilitates the formation of a CaCO_3_ shell. As shown in Fig. 1c, the TEM images of CUM show a significant core-shell structure in contrast with the morphology of pure UiO-66-NH_2_. And the particle sizes of CUM are increased to be 230 nm because of the formation of CaCO_3_ shell (Fig. 1d). As shown in the elemental mapping images of CUM (Fig. 1e), the mapping analysis indicates the presence of Ca, Mn C, O, N, and Zr elements, which further confirms the successful coordination of Mn and the CaCO_3_ shell coated onto the surface of the nanoparticles. Fig. 1f presents the X-ray diffraction (XRD) pattern of CUM, which correspond to the characteristic peaks of CaCO_3_ (JCPDS No.47-1743) and the simulated XRD pattern of UiO-66-NH_2_, indicating that CUM is successfully synthesized. In comparison with the peak positions of UiO-66-NH_2_, those of CUM remain unchanged, revealing the formation of single-atom Mn sites has a negligible effect on the crystal structure of UiO-66-NH_2_.

Furthermore, the elemental composition of the surface of CUM is analyzed using X-ray photoelectron spectroscopy (XPS). As shown in Fig. S2, the obvious Mn signals in XPS curve indicates the presence of Mn single-atoms within UiO-66-NH_2_ of CUM. In addition, the XPS spectra of CUM exhibits homogeneous distribution of the elements Ca, which is originated from the CaCO_3_ shell of the nanoparticles. Obviously, the Ca 2p spectrum is deconvoluted into two peaks: Ca 2p_3/2_ at 347.2 eV and Ca 2p_1/2_ at 350.8 eV, indicating the surface of UM is effectively coated with CaCO_3_ (Fig. 1g). Meanwhile, the high-resolution XPS profiles of Mn 2p are presented in Fig. 1h. The distinct Mn signal can be fitted into these peaks at approximately 653.1, 645.9, and 641.7 eV assigned to Mn 2p_1/2_, Mn sat, and Mn 2p_3/2_, respectively, confirming the formation of Mn single-atoms. As exhibited in Fig. 1i, the weight loss value (≈61.12 %) of CUM is much larger than that (≈51.37 %) of UM, suggesting that the designed Mn-based SAzymes have been successfully coated with CaCO_3_ shell. In addition, the Fourier transform infrared (FT-IR) spectrum is also an effective method to verify the successful synthesis of CUM. The FT-IR spectra of different samples are shown in Fig. 1j, indicating that these two samples (UM, and CUM) have similar characteristic peaks, which can be attributed to the stretching vibration of Zr-O and C=O at 3391 cm^−1^, 663 cm^−1^ and 1656 cm^−1^, originated from the framework of UiO-66-NH_2_. Following the introduction of calcium sources, the bands corresponding to C–O symmetric stretching vibrations at 1426 cm^−1^ and C–O bending vibrations at 869 cm^−1^ gradually intensified, indicating the progressive formation of the CaCO_3_ shell. Furthermore, the normalized UV–vis absorption spectra of UM and CUM exhibit a similar absorption band compared to that of UiO-66-NH_2_, indicating that their structures are based on UiO-66-NH_2_ (Fig. 1k). However, CUM also show significant absorption peaks of CaCO_3_ at 201 and 282 nm, indicating UM is successfully coated with CaCO_3_ shell.

### 3.2 The local electronic structure and coordination information of CUM

**Fig. 2:**
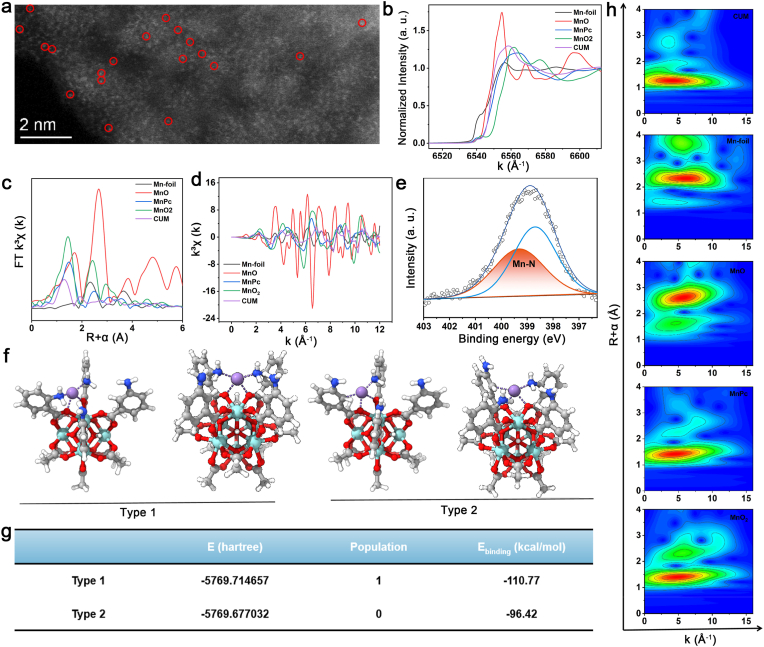
XAS and DFT of CUM SAzymes. (a) Spherical aberration-corrected TEM images of CUM. (b) Normalized XANES of the Mn K-edge spectra. (c) FT-EXAFS fitting curves at the R space for CUM. (d) K^3^χ(k) space spectra fitting curve of Mn-foil, MnO, MnO_2_, MnPc and CUM. (e) N 1s XPS spectra of the CUM. (f,g) View and energy data showing two types of single Mn oxide clusters in UiO-66-NH_2_, UiO-66-NH_2_ and Mn single-atom binding to the amino group. (h) WT-EXAFS plots of the Mn-foil, MnO, MnO_2_, MnPc and CUM. The O, N and Mn atoms are shown in red, blue and purple, respectively.

To further confirm the binding of a single Mn atom to UiO-66-NH_2_, aberration-corrected high-angle annular dark-field scanning TEM (AC HAADF-STEM), X-ray absorption near edge structure (XANES) and Fourier-transformed extended X-ray absorption fine structure (FT-EXAFS) experiments analyses are conducted. As shown in Fig. 2a, the individually dispersed bright dots enclosed by red circles in the AC HAADF-STEM image of the CUM corresponding to the single Mn atoms. The Mn K-edge in the XANES spectrum of the CUM deviates from the reference spectra of Mn-foil, MnO, MnO_2_, and MnPc, indicating a distinct valence state of the single Mn atom (Fig. 2b). Meanwhile, the presence of a single Mn atom in the CUM can be further confirmed by the FT-EXAFS analysis. In contrast to the characteristic peaks of Mn–Mn bond observed in the Mn foil, the Mn peaks of CUM are matched with the Mn–N bond of the MnPc, indicating that the coordinated Mn ions exist as single-atom sites within the CUM (Fig. 2c). Moreover, additional evidence supporting the Mn-based single-atom sites in CUM is provided through the EXAFS fitting curves in both R space and K spac shown in Fig. 2d, Fig. S3 and Table S2. EXAFS fitting and quantitative analysis reveal that the coordination number of the single Mn atoms in the CUM is 4, consistent with that of Mn-N in MnPc. N 1s XPS analysis of CUM is further conducted to investigate the formation of single-atom Mn sites. Due to the fitting curves, the presence of Mn-N bonding of CUM provides evidence for the successful incorporation of Mn with the functional groups of UiO-66-NH_2_ (Fig. 2e and S4). Additionally, the Mn 2p peak observed at 641.7 and 653.1 eV in CUM, along with the detectable Mn-N/Mn-O peaks in the Mn 2p region of the CUM XPS spectrum (Fig. 1h), suggests the formation of single-atom Mn sites.

Density functional theory calculations are performed using the ORCA 6.0.0 software with a Gaussian interface [31,32], revealing two distinct local coordination configurations of the single Mn atom: one involving coordination with amino groups from organic ligands, and the other involving coordination with an O atom derived from Zr clusters in the UiO-66-NH_2_ framework. (Fig. 2f). Based on the spatial distribution of the amino groups in the ligands, the Type 1 local coordination model is constructed, in which a single Mn atom coordinates with two adjacent amino groups and two neighboring O atoms. In contrast, the Type 2 structure is unlikely to exist due to its extremely high energy (Fig. 2g). Furthermore, the wavelet transformation (WT) plots of the CUM, Mn-foil, MnO, MnO_2_ and MnPc are drawn to visualise the corresponding EXAFS spectra based on the *k* and *R* spaces values (Fig. 2h). Based on the above experiments, the Mn single-atom nanozyme is confirmed to exist and demonstrates considerable potential for application in tumor therapy.

### 3.3 Ca^2+^ release and the single-atom nanozyme performance of the CUM

**Fig. 3:**
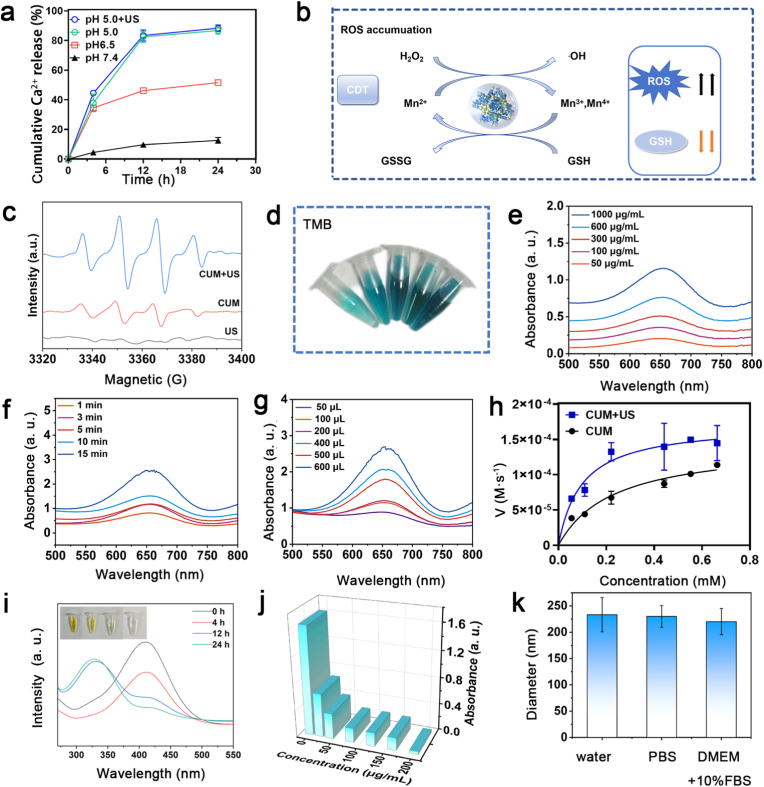
Characterizations of catalytic activity of CUM. (a) Detection of the *in vitro* release of Ca^2+^. (b) Schematic diagram of GSH depletion and ∙OH generation of CUM. (c) ESR spectra of ∙OH trapped by DMPO. (d, e) UV spectroscopy of different groups when using TMB probes for detecting POD-like activity (TMB + H_2_O_2_ + CUM groups with 50, 100, 300, 600 and 1000 μg/mL of TMB, respectively). (f, g) The ox-TMB absorbance by ·OH, generated from reactions of under US time and H_2_O_2_ concentrations. (h) Michaelis−Menten kinetic analysis of CUM/CUM + US + H_2_O_2_ under different conditions. Analysis of GSH consumption caused by CUM at different time periods (i) and at different concentrations (j). (k) The hydration particle of CUM after incubation with water, PBS and DMEM+10 % foetal bovine serum (FBS).

The Ca^2+^ release and single-atom nanozyme activity of CUM are systematically investigated, providing critical evidence to support subsequent antitumor experiments. Firstly, the pH-responsive dissociation of CUM and release of calcium ions are evaluated by dissolving the nanoparticles in buffer solutions of varying pH values. The calcium content in the supernatants was measured at various time points using Inductively Coupled Plasma Mass Spectrometry (ICP-MS) to generate calcium release profiles over time under different pH conditions. As shown in Fig. 3a, the concentrations of Ca^2+^ ions increased markedly as the pH reduces from 7.4 to 6.5 and 5.0, suggesting that CUM can be efficiently released within the TME, thereby exposing their internal components for targeted tumor therapy. To further elucidate the antitumor performance of CUM, Fig. 3b presents a schematic diagram illustrating the underlying mechanism of the single-atom nanozymes. The electron spin resonance (ESR) technique is also an effective method for investigating the generation of ·OH. As shown in Fig. 3c, the ESR signals of CUM with H_2_O_2_ solution represents 1:2:2:1 (a characteristic ESR signals of ·OH), indicating it possesses excellent POD-like activity. Under US irradiation, the CUM exhibits the higher POD-like activity compared to other conditions, indicating that the US cavitation effect enhances the concentration of H_2_O_2_. And ∙OH was not generated in the group of US treatment of H_2_O. TMB is employed as an indicator to assess the enzyme-like activity of CUM, and a corresponding increase in ox-TMB absorbance at 650 nm is observed. The absorption peak observed in the UV–vis spectra at varying concentrations of CUM exhibits a concentration-dependent trend, which can be attributed to the progressive enhancement of their enzyme-like activity with increasing nanoparticle concentration (Fig. 3d and e). Consistent results are also obtained when the duration of ultrasound treatment (Fig. 3f) and the concentration of H_2_O_2_ (Fig. 3g) are varied. These findings suggest that these nanozyme catalysts, particularly the POD, follow the Michaelis-Menten kinetics, as described by the following equation:

$$V=\frac{V_{\max}S}{V_{\max}+S}$$

The reaction initiation velocity (*V*) is determined by the maximum reaction velocity (*V*_max_), the substrate concentration ([*S*]), and Michaelis constant (*K*_m_). Generally, a lower Km value indicates a higher enzyme affinity for the substrate. As shown in Fig. 3h and S5, under the same experimental conditions, the Vmax value of US-irradiated CUM is 1.7 × 10^−4^ M∙s^−1^, higher than that of CUM (1.4 × 10^−4^ m∙s^−1^). The change of Km values differs from the variation in Vmax, in which Km of CUM (0.23 mM) is two times that of US irradiation (0.1 mM). These results indicate that the CUM exhibits enhanced catalytic kinetics and stronger substrate affinity for intracellular H_2_O_2_ under US irradiation. At the same time, compared with similar nanozymes, it showed good catalytic ability, and under the assistance of US, the catalytic ability was stronger. [33,34]. After glutathione (GSH) is co-incubated with the reacted CUM, the absorbance intensity of GSH at 412 nm gradually decreased, implying that intracellular GSH will be consumed under the high oxidative state associated with single Mn atom sites after reaction with H_2_O_2_ (Fig. 3i and j). Because Mn^2+^ can be oxidized by H_2_O_2_ to generate ·OH and hypervalent Mn^3+^/Mn^4+^ species [35]. Subsequently, GSH mediates the reduction of Mn^3+^/Mn^4+^ back to Mn^2+^, resulting in the formation of oxidized glutathione (GSSG), thereby initiating the cascade POD-like activity and causing GSH depletion. Given the importance stability in physiological environment for biomedical applications, the stability test is also performed on the CUM. Following incubation in water, PBS, and DMEM supplemented with 10 % fetal bovine serum (FBS), no significant change in particle sizes is observed (Fig. 3k), suggesting good colloidal stability. Overall, owing to its favorable pH responsiveness and excellent catalytic performance under US irradiation, CUM is expected to be well-suited for *in vitro* and *in vivo* experiments.

### 3.4 In vitro anti-tumor effect of CUM

**Fig. 4:**
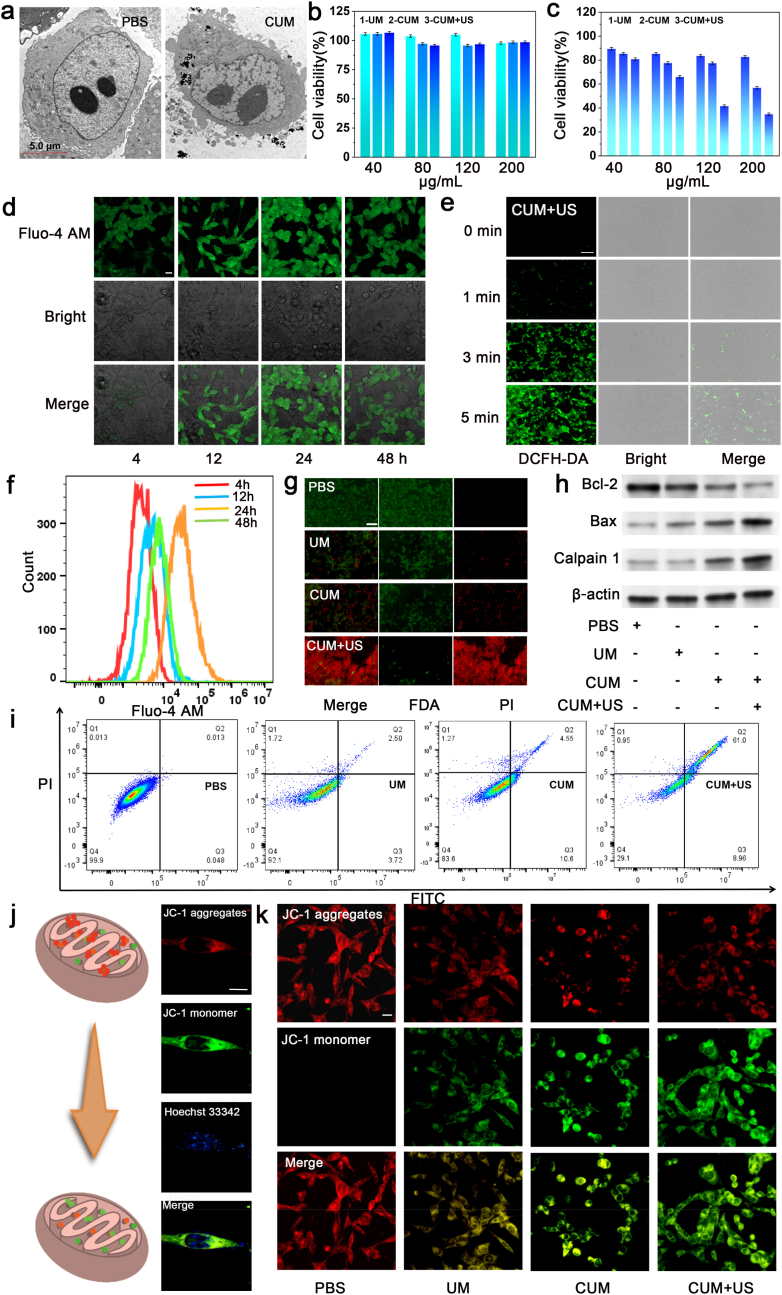
*In vitro* analysis of the anti-tumor effect of CUM. (a) Representative bio-TEM images of 4T1 cells incubated with different conditions. Cell viability analysis of (b) L929 and (c) 4T1 cells treated with UM, CUM and CUM + US. Detection of (d) the intracellular Ca^2+^ release by Fluo-4 AM (Scale bar = 20 μm) and (e) ROS production using DCFH-DA (Scale bar = 100 μm). (f) Ca^2+^ intensity detected by flow cytometry at different time periods. (g) Live/dead-stained images of 4T1 cells s treated with PBS, UM, CUM and CUM + US. Scale bar = 100 μm. (h) Western blot for Bax, Bcl-2 and Calpain proteins after treatment with PBS, UM, CUM and CUM + US. (i) Flow cytometric analysis of apoptotic cells after incubation with PBS, UM, CUM and CUM + US. (j) Diagrammatic sketch of JC-1 staining; Scale bar = 10 μm. (k) JC-1 staining images in 4T1 cells after treatment with PBS, UM, CUM and CUM + US. Scale bar = 20 μm. The power density of the ultrasound is 1.5 W/cm^2^, and the irradiation time is 3 min.

Subsequently, the antitumor effect of CUM is evaluated in the 4T1 murine breast cancer cell line. As shown in Fig. 4a, the uptake of CUM by the cytoplasm of tumor cells is observed using bio-TEM, which indicated that the amount of CUM in the cytoplasm increased progressively over time (Fig. S6). Then, the biosafety and toxicity *in vitro* of CUM should be addressed. At first, L929 cells are exposed to varying concentrations of CUM for 24 h, after which its biosafety is assessed using the standard MTT assay. Even at concentrations as high as 200 μg/mL with US irradiation, CUM demonstrates high biocompatibility toward normal cells, as indicated by the minimal cytotoxicity observed (Fig. 4b). However, CUM + US shows a dose-dependent inhibitory effect on 4T1 cells in Fig. 4c, with a median inhibitory concentration (*IC*_50_) of approximately 120 μg/mL (Fig. S7a). In contrast with UM group, the cells incubated with CUM show high cytotoxicity for 4T1 cells, verifying that CUM not only possesses POD-like activity to generate ·OH but also induces disorders of intracellular and extracellular calcium ions regulation to achieve synergistic anti-tumor effect. Especially, the cell viability of the group treated with a high concentration of CUM after US irradiation decreases to approximately 35 %, indicating that the cavitation effect of US can enhance the nanocatalytic therapy of the SAzymes we designed. Meanwhile, the US-alone treatment group did not cause significant damage to the cells (Fig. S7b and c). Subsequently, the Ca^2+^ ions are released in the TME through the degradation of the CaCO_3_ shell in the CUM nano-system confirmed using Fluo-4 AM (a kind of Ca^2+^ fluorescent probe). The Fluo-4 AM is a membrane-sensitive, lipophilic anion dye that binds to the cell membrane, which has an excitation wavelength of 494 nm and an emission wavelength of 516 nm. The probe exhibits minimal fluorescence in the absence of Ca^2+^ binding, and demonstrated a significant increase in fluorescence intensity after integration with the dissociative Ca^2+^. As shown in Fig. 4d and S8, the green fluorescence signal in the CLSM images of CUM, as well as the fluorescence intensity quantified by ImageJ analysis, gradually increased over time. The results demonstrate that the concentration of released Ca^2+^ increased progressively over time because of CUM decomposition, suggesting effective cellular uptake, which can be attributed to appropriate particle sizes and favorable pH-responsive properties that induce calcium-mediated cell death. Furthermore, to measure ROS production of CUM in 4T1 cells, DCFH-DA is employed to assess intracellular ROS levels following different exposure durations to US irradiation, which DCFH-DA will be oxidized to fluorescent DCF by the generated ·OH originated from CUM in the TME. As shown the fluorescence images in Fig. 4e–S9 and S10, the DCF signals are become stronger with the CUM-incubated time increases, suggesting that the single Mn atoms coordinated in the CUM trigger robust POD-like activity to induce ·OH generation in the TME and thereby contributing to oxidative stress-mediated cell damage [36]. Consistent with the CLSM results obtained from Fig. 4d, the flow cytometry analysis in Fig. 4f reveals that the fluorescence intensity of Ca^2+^ changed following different incubation times with CUM, suggesting a time-dependent enhancement of pH-responsive properties of CUM. The glutathione assay kit is utilized to estimate the GSH depletion of CUM *in vitro*. Compared with the non-treatment group, the endogenous GSH concentrations of 4T1 cells are significantly decreased after incubating with CUM (Fig. S11), suggesting that CUM effectively consumes GSH in TME, laying the foundation for the subsequent CDT process. Immediately afterward, 4T1 cells are co-stained with fluorescein diacetate (FDA) and propidium iodide (PI) under various treatment conditions to further assess the extent of cell death following CUM intervention (Fig. 4g and S12a). While the cells in the control group exhibit a green fluorescence, those US-treatment group does not exhibit significant differences, those UM-treatment group displays partial red fluorescence, indicating the presence of dead 4T1 cells because of the therapeutic effect of the designed Mn-based SAzymes. And the images of CUM group exhibits the increased red fluorescence signals attributed to the synergistic effect of calcium-mediated cell death and nanocatalytic therapy. Especially in the group treated with CUM under US irradiation, almost all cells are stained red to prove that the majorities of the cells have lost viability, indicating that the enzymatic catalysis performance of CUM can be promoted by the cavitation effect of US. Simultaneously, as displayed in Fig. 4h, western blot analysis demonstrated that both the levels of apoptosis and the expression of calcium-related proteins are markedly elevated in the CUM group compared to those in the control and UM group. And after CUM combination with exposure to US radiation, the therapeutic effect is obvious enhanced observed by the intensity of the apoptosis. In addition, the flow cytometry analysis and JC-1 staining further suggests that CUM influence intracellular apoptosis and the expression levels of calcium-associated proteins, thereby exerting anti-tumor effects. As shown in Fig. 4i and S12b, the flow cytometry results demonstrating cellular apoptosis are consistent with the findings obtained from live/dead staining in Fig. 4g. To further validate the role of Ca^2+^ overload in cell death, the fluorescent probe JC-1 is employed to detect mitochondrial membrane potential changes, which can also be served as an indicator of cellular oxidative damage. After apoptosis, the JC-1 fluorescence signals of the treated cells transition from red to green, where the red and green emissions represent the JC-1 monomer and its aggregates, respectively. As exhibited in Fig. 4j, it indirectly reflects ROS-induced oxidative damage and the role of Ca^2+^ overexpression by observing the mitochondrial status around the nucleus. The experimental groups are as follows: the control group, and the treatment group with US, UM, CUM, and CUM with US irradiation. As shown in Fig. 4k and S12c, compared with the PBS group, the red fluorescence intensity decreases, while the green fluorescence intensity increases following treatment with UM, suggesting the POD-like performance of the designed Mn-based SAzymes. After coating with CaCO_3_ shell, the CUM-treated group shows enhanced green fluorescent signals, which can be attributed to the pH-responsive release of Ca^2+^ to induce mitochondrial dysfunction. The significant changes were not observed in the US group. Moreover, under the assistance of US radiation, the green fluorescence signals are further increased due to the improved enzymatic catalytic activity of CUM, which is promoted by cavitation effect of US. Therefore, these results demonstrate that CUM exhibits a significant *in vitro* anti-tumor effect following ultrasound radiation treatment.

### 3.5 Imaging assessment of CUM

**Fig. 5:**
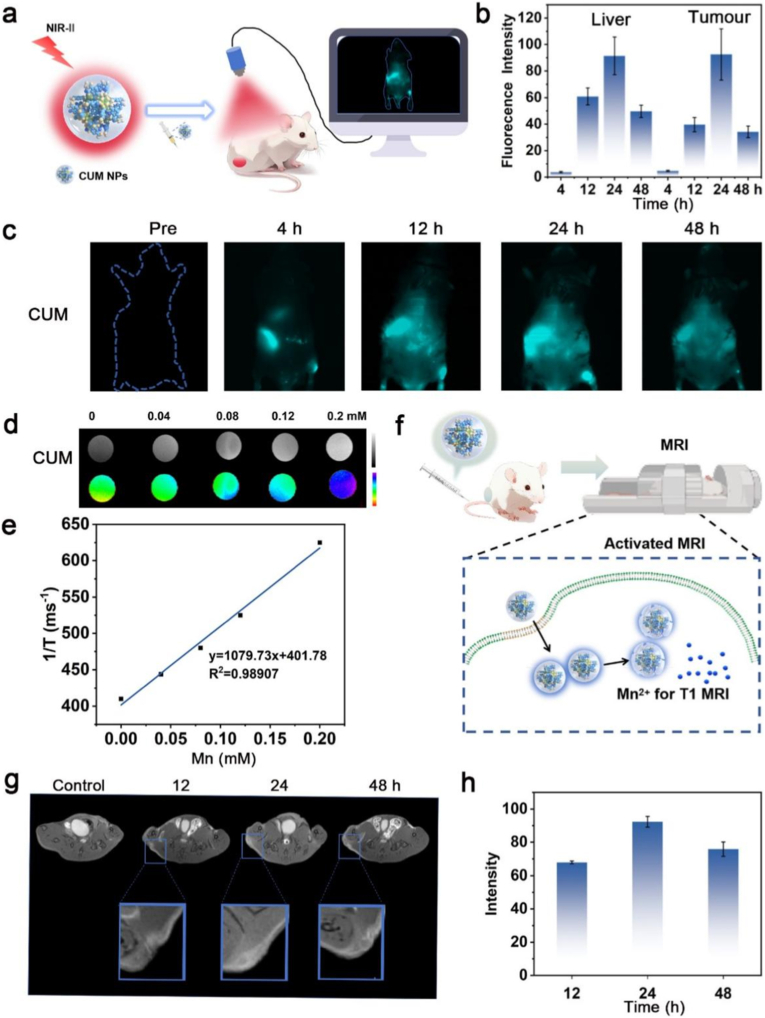
Imaging assessment of CUM *in vitro* and *in vivo*. (a) The schematic diagram of NIR-II fluorescence imaging of CUM under 808 nm laser irradiation. (b) The NIR-II fluorescence intensity analysis of the liver and the tumor site after different post-injection time of CUM. (c) The NIR-II fluorescence images of the tumor-bearing mice after post-injection of CUM. (d) *T*_1_-weighted MR intensities of CUM at different Mn concentration. (e) The curve of 1/*T*_1_ versus CUM at elevated Mn concentration. (f) The schematic diagram of *T*_1_-weighted MRI performance from CUM. (g) MR *T*_1_ weighted image of tumor-bearing mice (region enveloped by the blue dotted line) at 12 h, 24 h and 48 h post-injection of CUM and (h) Intensity analysis of tumor image.

Encouraged by the promising outcomes from the *in vitro* studies, the *in vivo* anti-tumor therapeutic efficacy of CUM is further evaluated. Therefore, the bio-imaging assessment of CUM should be prioritized to determine the optimal treatment timing. The NIR-II fluorescence imaging monitoring of 4T1-bearing mice tail-intravenous injection of CUM-labeled with ICG is performed (Fig. 5a), which has been applied for precise tumor diagnosis and treatment, stem cell tracking, and targeted drug delivery [[37], [38]]. As shown in Fig. 5b and c, CUM is found to accumulate at the tumor site by the Enhanced Permeability and Retention (EPR) effect, with the accumulation increasing progressively over time. After 24 h post-injection of CUM-labeled with ICG, the ICG signals at the tumor site reached a peak and then gradually declined. Then, the fluorescence signals almost disappear in the tumor tissues until 48 h, confirming that the CUM has been metabolised. Furthermore, the *in vivo* MRI performance of the CUM demonstrates its favorable biodistribution profile, which the coordinated Mn^2+^ functions as a contrast agent that enhances the visibility of tissues in *T*_1_-weighted MRI [36]. At first, the *T*_1_ signal intensity of the CUM increases significantly with the increasing concentration of Mn^2+^ (ranging from 0 to 0.2 mM). At 12, 24, and 48 h following CUM injection, the MR *T*_1_-weighted image signals in tumor-bearing mice initially increased gradually and subsequently decreased after 48 h (Fig. 5d–f). Then, the MRI performance of the CUM is tested *in vivo* exhibited in Fig. 5g and h. When the post-injection time is extended, the CUM are gradually degraded. This accumulation pattern of CUM closely correlate with the NIR-II fluorescence imaging of tumor, which shows a progressive increase followed by a gradual decline over time. Based on this observation, the optimal time window for *in vivo* intervention is determined, and subsequent animal experiments are carried out accordingly.

### 3.6 In vivo systemic anti-tumor potency and bioinformatics analysis of CUM

**Fig. 6:**
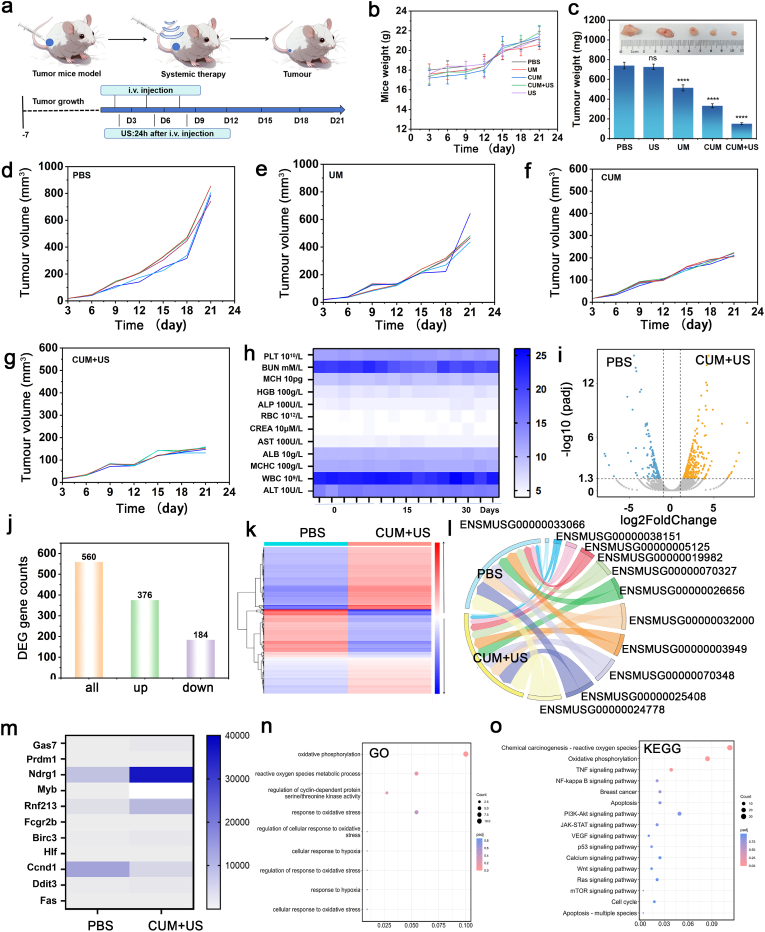
Systemic antitumor potency and bioinformatics analysis of CUM *in vivo*. (a) Schematic diagram of the establishment of the 4T1 tumor-bearing mouse model and the treatment of CUM + US. (b) Body weight of mice in the PBS, US, UM, CUM and CUM + US. (c) tumor weights of tumor-bearing mice in different treated groups. The tumor volume of 4T1 tumor-bearing mice after treated with PBS (d), UM (e), CUM (f) and CUM + US (g). (h) Hematological indexes of mice after intravenous injection with CUM. (i) Volcano plot analysis of the PBS *vs* CUM + US. (j, k) Differential gene expression analysis between the PBS and CUM + US. (l, m) Analysis of cancer-related genes among the differential genes between the PBS and CUM + US. Enrichment analysis of the GO database (n) and the KEGG database (o). ∗∗∗∗*P* < 0.0001.

As shown in Fig. 6a, the mice subcutaneous 4T1 tumor-bearing models are established to evaluate the *in vivo* anti-tumor efficacy of CUM. Firstly, the mice are randomly assigned to four groups: the control group, the US group, the UM group, the CUM group, and the CUM combined with US irradiation (CUM + US) group. On days 2, 4, and 8, CUM are administered to the mice via tail vein injection, and then the CUM + US-treated mice are irradiated by US for 3min after 24 h of post-injection. During the experiment, the body weight and tumor volume of the mice in each group are measured every three days to assess the anti-tumor efficacy. In Fig. 6b, it can be observed that the mean weight increased at 21 days in the experimental groups are comparable to that in the PBS control group, indicating CUM shows high biocompatibility *in vivo*. Fig. 6c demonstrates that the tumor weight in the CUM group is significantly lower than that in the control group, and the reduction is further augmented following US treatment, verifying the synergistic effect of calcium-mediated cell death and nanocatalytic therapy of CUM under US irradiation. In addition, the CUM + US group demonstrates significantly greater tumor growth inhibition compared to the PBS group and other treatment groups (Fig. 6d–g and Fig. S13). As shown in Fig. S14, the hematoxylin and eosin (H&E) and TUNEL staining results reveals that tumor cells in the PBS group exhibited a compact and dense morphology with minimal signs of apoptosis, whereas the experimental group displays obviously damaged cellular structures and extensive apoptotic changes. Moreover, the haematological indicators of the mice remain within normal ranges after intravenous administration of CUM, suggesting that these nanoparticles can not induce haematological toxicity in the mice (Fig. 6h). To further clarify these key indicators, blood biochemical analyses were conducted on mice from different treatment groups (PBS, US, UM, CUM, CUM + US), as illustrated in Fig. S15. The results showed that ALT, AST, BUN, and CREA levels remained within normal ranges. In conclusion, CUM demonstrates excellent biocompatibility. Furthermore, the normal tissues extracted from mice treated with CUM show no significant differences compared to those in the control group, indicating the biosafety of the designed SAzyme (Fig. S16 and S17). In the body circulatory system, the liver, as important organ, is generally considered the primary site for accumulation and elimination of the nanoparticles. Likewise, using inductively coupled plasma emission spectroscopy (ICP-OES) technology, CUM bioavailability data were obtained from tumor-bearing mouse tissues at different intravenous injection time points. As shown in Fig. S18a and b, Ca and Mn concentrations in the tumor site gradually increased over time, followed by stable metabolism within 72 h.

To elucidate the underlying therapeutic emechanism of CUM, RNA sequencing analysis is conducted. Bioinformatics analysis is performed to compare the expression profiles of relevant RNA in 4T1 cells between the PBS and CUM + US groups, aiming to elucidate the underlying molecular mechanisms. As shown in Fig. S19, among the numerous genes expressed, the Venn diagram revealed that 11,206 genes are co-expressed in both the CUM + US and control groups. In contrast with control group, the volcano plot of CUM + US group exhibits a total of 560 differentially expressed genes (DEGs) are identified, with 376 genes upregulated and 184 genes downregulated (Fig. 6i and j), which the result is consisted with the data obtained from the heatmap analysis shown in Fig. 6k. The chord plot and heatmap reveals that Ccnd1 genes associated with breast cancer are significantly down-regulated in the CUM + US group (Fig. 6l, m, and S20). Furthermore, bioinformatics analysis exhibited in Fig. 6n revealed that the major biological processes enriched in CUM + US-treated cells are associated with the response to regulation of the inflammatory response, and ROS metabolism. According to the Kyoto Encyclopedia of Genes and Genomes (KEGG) database, CUM is involved in oxidative phosphorylation, ROS metabolism, as well as the signaling pathways of TNF, p53, and calcium ions **(**Fig. 6o). Meanwhile, auxiliary experiments were conducted at the protein level for both p53 and TNF-α, confirming that CUM intervention affects protein expression. **(**Fig. S21). Therefore, CUM combined with US irradiation can combat cancer through the coordinated regulation of multiple pathways, thereby achieving a synergistic therapeutic effect through calcium-mediated cell death and nanocatalytic therapy.

## 4 Conclusions

In summary, the CaCO_3_-mediated Mn-based SAzymes have been developed in this study. The excellent enzymatic catalysis performance (POD-like activity) CUM is originated from the Mn-based single-atom sites, and can be enhanced by the cavitation effect under US irradiation. Notably, the coated CaCO_3_ shell of CUM degrades within the TME, enabling pH-responsive release of Ca^2+^and thereby inducing calcium-mediated cell death. Furthermore, the optimal treatment time and biodistribution of CUM is confirmed by multiple bio-imaging assays (NIR-II fluorescence imaging and *T*_1_-weighted MRI). And the therapeutic emechanism of CUM upon US irradiation is evaluated by RNA sequencing analysis, which CUM exerts significant effects on the normal biological functions of tumor cells by inducing calcium-mediated cell death and activating apoptosis-related signaling pathways. Therefore, this study highlights the synergistic therapeutic application of CUM in overcoming the physiological barriers within the TME, thereby enabling more effective cancer therapy.

## Funding

Financial support was received from the National key research and development plan (NO. 2023YFB3810002), National Natural Science Foundation of China (82302258, 32101112, U22A20348), the Taishan Scholar Project of Shandong Province (tsqn202211249), the Natural Science Foundation of Shandong Province (ZR2022QB106, ZR2022MB104, and ZR2023QB059), the Youth Innovation Team of Shandong Provincial Higher Education Institutions (2023KJ295).

## CRediT authorship contribution statement

**Xiao Wang:** Writing – original draft, Investigation, Data curation. **Bingyu Xu:** Writing – original draft, Investigation. **Fang Li:** Writing – original draft, Validation. **Zhao Wang:** Writing – review & editing, Supervision, Methodology, Conceptualization. **Quanxiang Han:** Writing – review & editing, Visualization, Methodology. **Yuxiao Li:** Writing – original draft, Validation, Data curation. **Yen Leng Pak:** Writing – review & editing, Validation. **Yurong Guo:** Writing – review & editing, Data curation. **Yingying Jing:** Writing – review & editing, Investigation. **Xing Gao:** Writing – review & editing, Validation. **Lei Yu:** Writing – review & editing, Supervision, Methodology. **Jibin Song:** Writing – review & editing, Supervision.

## Declaration of competing interest

The authors declare that they have no known competing financial interests or personal relationships that could have appeared to influence the work reported in this paper.

## Data Availability

Data will be made available on request.
