# Supplementary material for: Engineered calcium carbonate-modiated SAzymes with Mn-based single-atom sites for ultrasound-enhanced nanocatalytic therapy
Source: Mater Today Bio. 2025 Oct 30;35:102463. doi: 10.1016/j.mtbio.2025.102463 (PMC12639588; doi:10.1016/j.mtbio.2025.102463)

Supporting Information

[Engineered calcium carbonate-mediated SAzymes with Mn-based single-atom sites for ultrasound-enhanced nanocatalytic therapy](https://www.x-mol.com/paperRedirect/1920997640681431040)

**Experimental section**

*Validation of GSH Depletion at the Cellular Level*

4T1 cells were cultured with completed DMEM medium (10% fetal bovine serum, 100 IU/mL penicillin, and 100 μg/mL streptomycin) in a 37°C incubator with an air atmosphere containing 5% CO_2_. For GSH detection *in vitro*, the 4T1 cells are incubated into 6-well culture plates (1×10^4^ per well, n = 3) and then treated with CUM (200 μg/mL). US power is set as 1.5 W/cm^2^, 3 min(US: 1.0 MHz, 20% duty cycle). Then, according to the manufacturer’s instructions, the GSH contents in these treated cells are tested by using the GSH assay kits (Shanghai Jingkang Biotechnology Co., Ltd, W96).

*IC_50_ analysis*

4T1 cells were pre-cultured in 96-well plates one day prior and maintained for 16-18 hours. CUM solutions with varying concentrations (0, 40, 60, 80, 100, 120, 140, 160, 180, 200 μg/mL; US, 1.5 W/cm², 3 min) were pre-mixed and co-incubated with the cells. Subsequent procedures followed those established for MTT assays, with final detection performed using a microplate reader and analysis conducted via GraphPad Prism 8.0 software.

*ICP-OES analysis*

At different times postinjection, the above mice were sacrificed, and major organs were collected and dehydrated. The dehydrated organs are treated with concentrated nitric acid. ICP-OES was used to measure the biological distribution of Ca and Mn in major tissues at different times after injection.


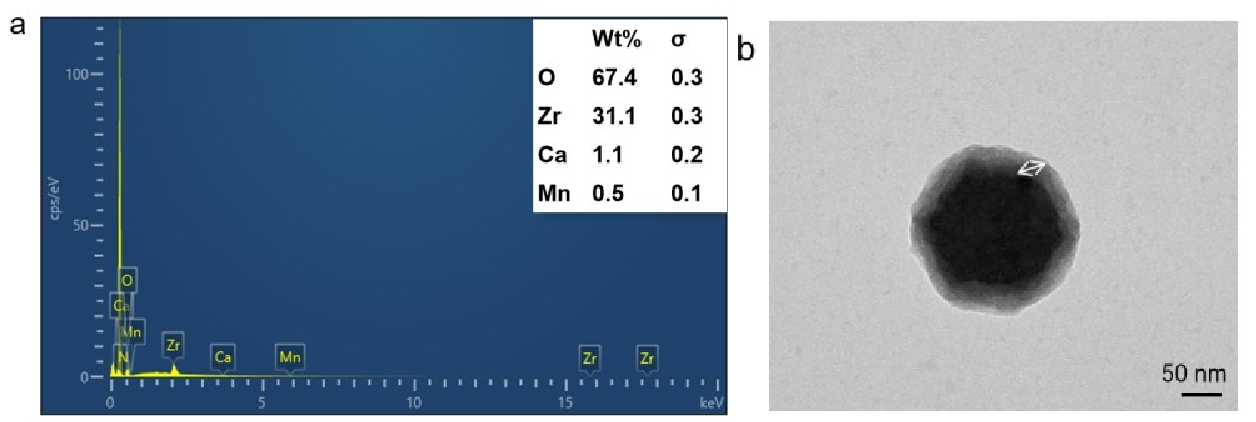


**Fig. S1. (a)** EDS analysis of CUM under SEM. (b)TEM image of CUM.

As shown in the figure below, XRD peak patterns reveal CaCO_3_ coating, while EDS analysis demonstrates Ca element distribution. However, due to the predominant presence of oxygen (O) and other elements, Ca is relatively scarc(Fig. S1a). At the same time, further observation through scanning electron microscopy showed that there was a coating phenomenon in the outermost layer, which further proved the formation of CaCO_3_ shell (Fig. S1b).


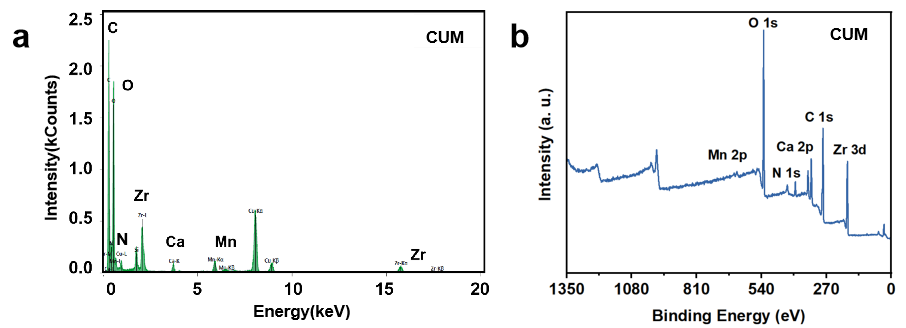


**Fig. S2.** Element proportion of CUM.


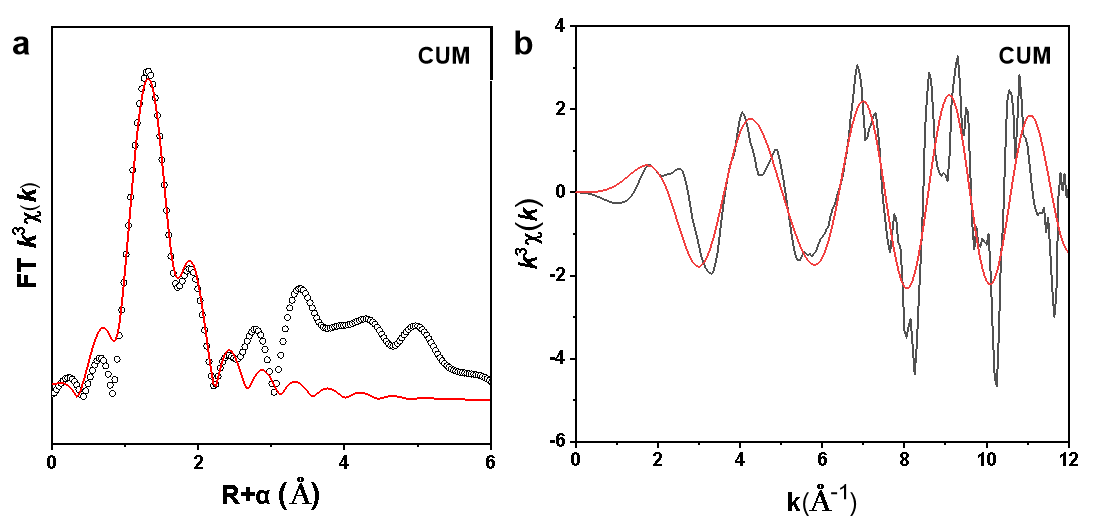


**Fig. S3.** FT-EXAFS fitting curves at R (a) and κ (b) space of Mn K-edge for CUM.

**
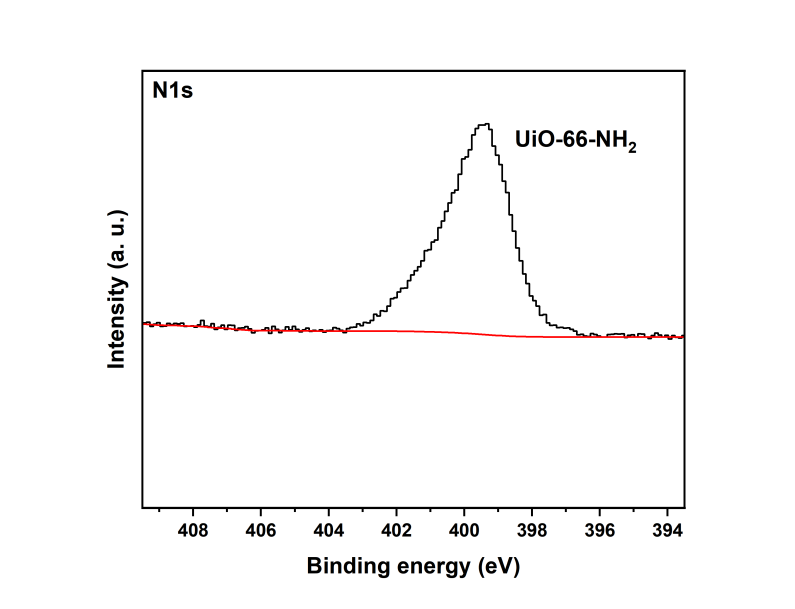
**

**Fig. S4.** XPS spectra of N 1s of UiO-66-NH_2_.


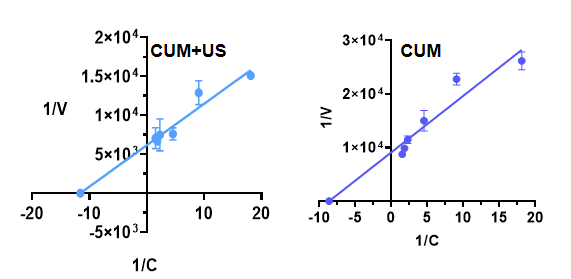


**Fig. S5.** Michaelis−Menten kinetic analysis of CUM/CUM+US + H_2_O_2_ under logarithmic form.


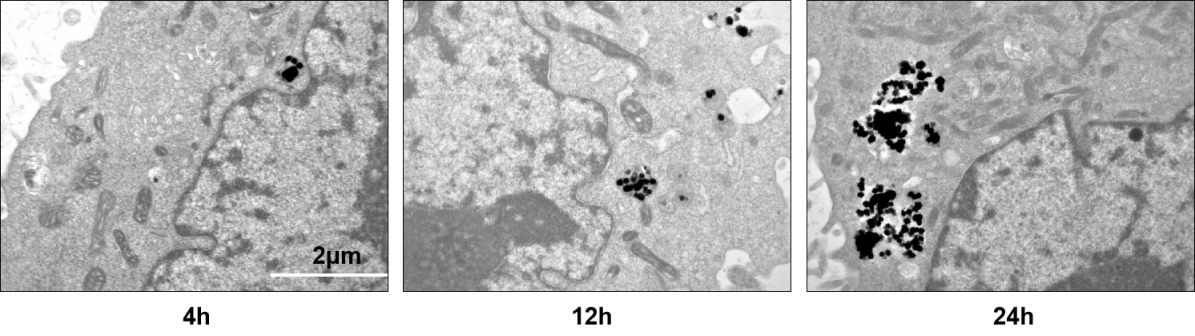


**Fig. S6.** The bio-TEM images of the cells treated with CUM under different incubation times.


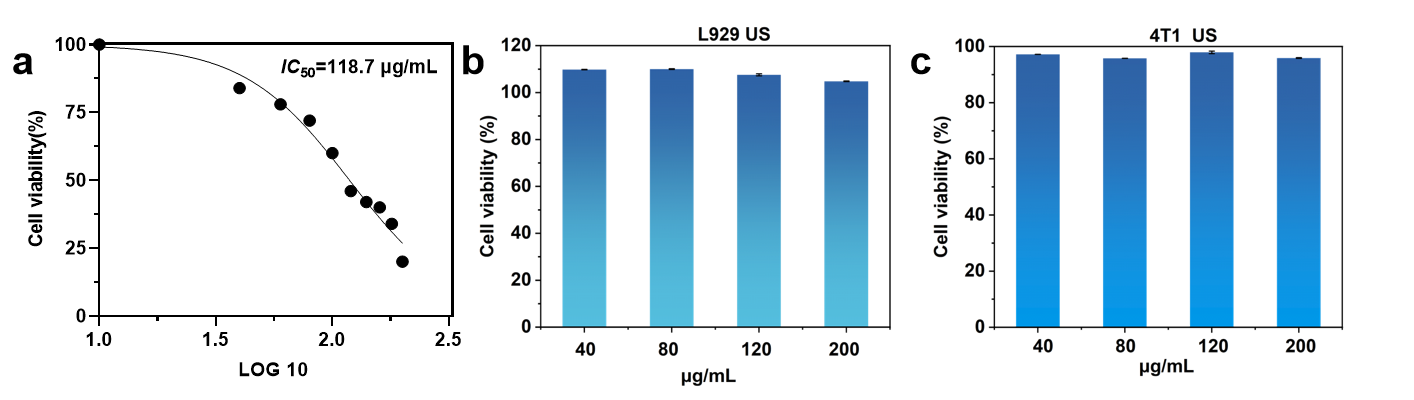


**Fig. S7.** (a) *IC*_50_ analysis of CUM treated cells.(b, c)Analysis of cell viability after US alone.


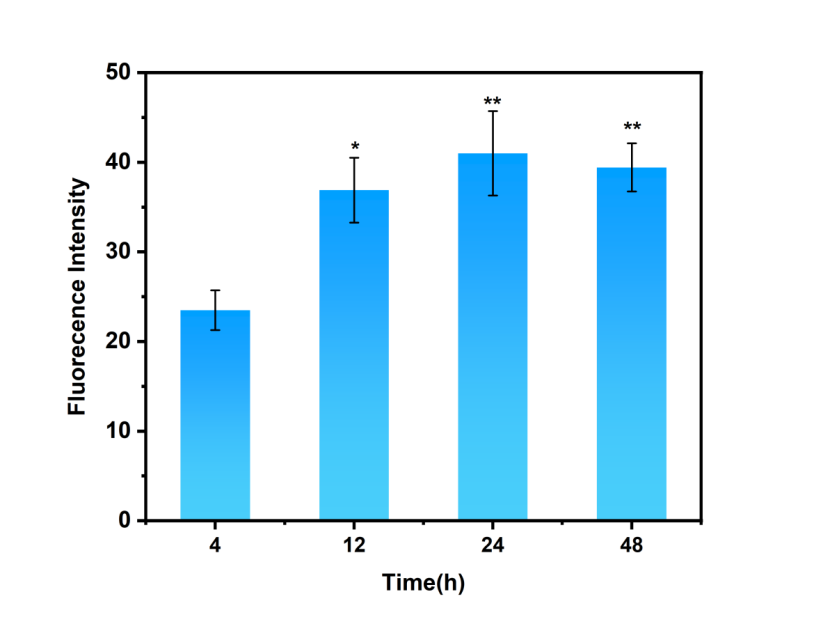


**Fig. S8.** The fluorescence intensity of analysis of the Ca^2+^. **P* < 0.05, ***P* < 0.01.


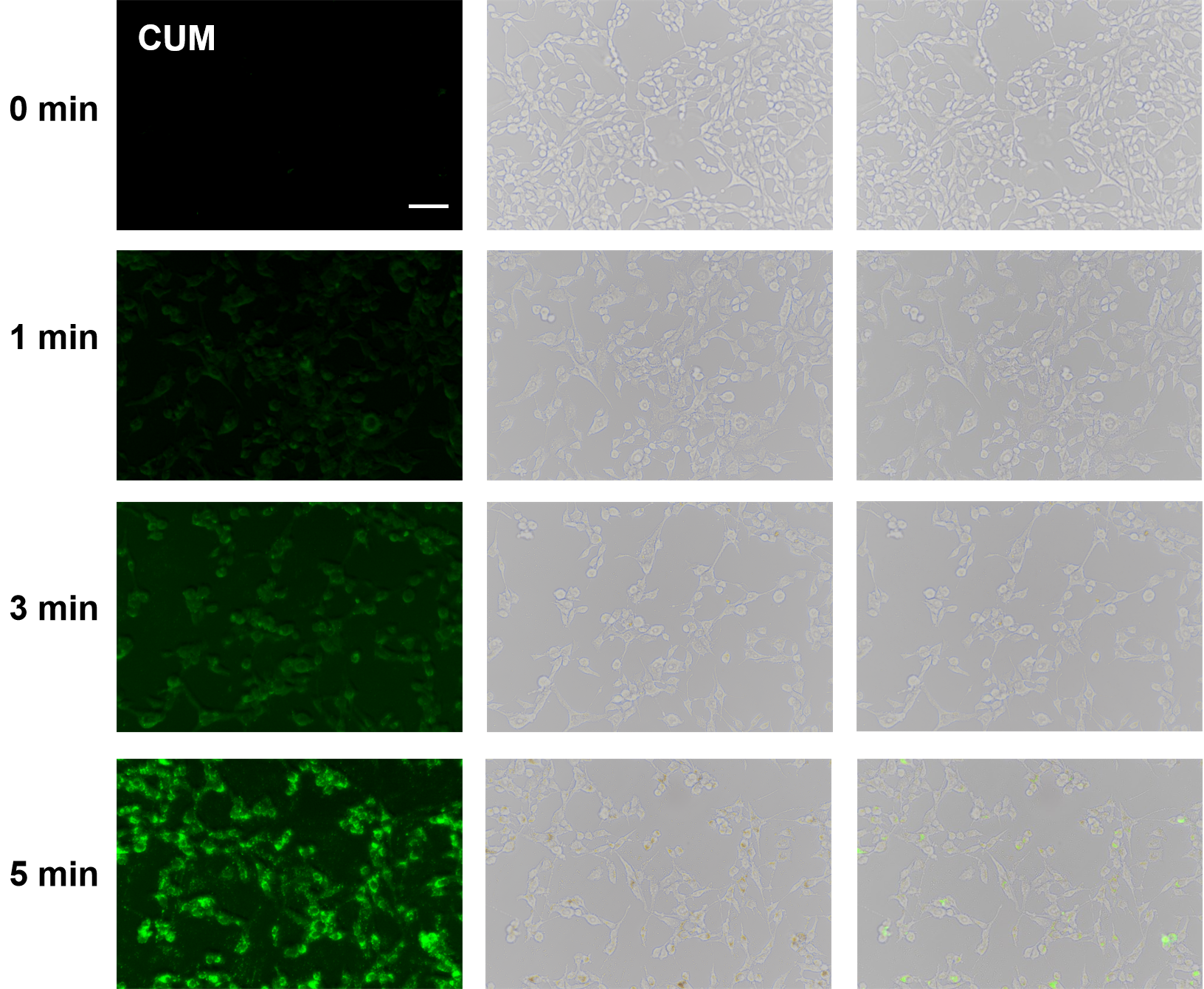


**Fig. S9.** The generation of ROS in intracellular CUM group was detected by DCFH-DA. Scale bar: 100 μm.


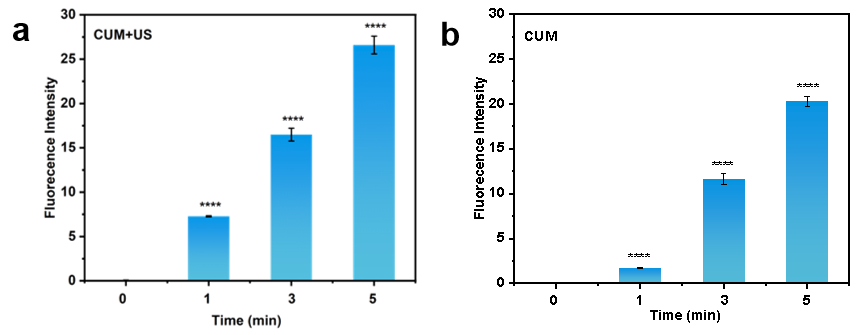


**Fig.S10.** The fluorescence intensity of analysis of the generation of ROS in intracellular CUM and CUM+US group.*****P* < 0.0001.


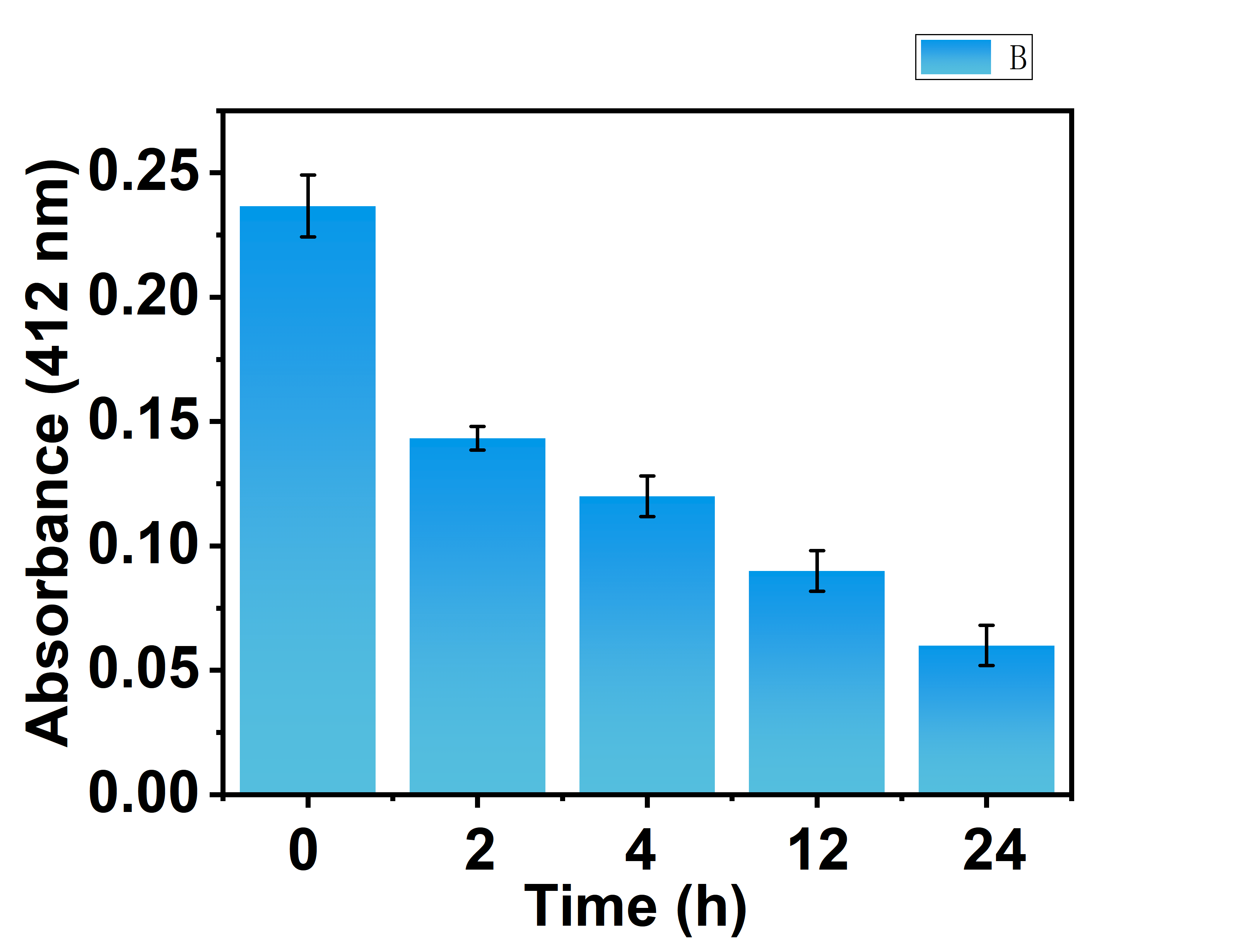


**Fig.S11.** 4T1 cells co-incubated with CUM to monitor glutathione depletion under US intervention.


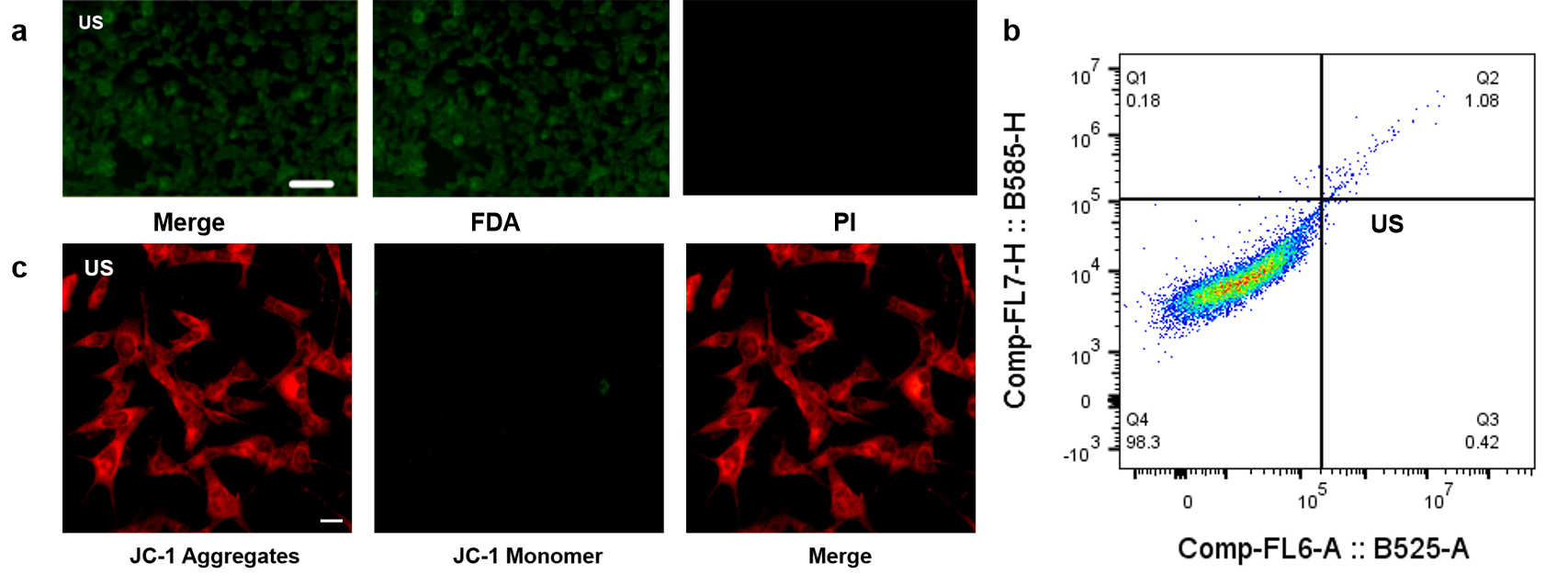


**Fig.S12.** (a) Live/dead-stained images of 4T1 cells s treated with US. Scale bar: 100 μm. (b)Flow cytometric analysis of apoptotic cells after incubation with US.(c)JC-1 staining images in 4T1 cells after treatment with US. Scale bar: 20 μm.


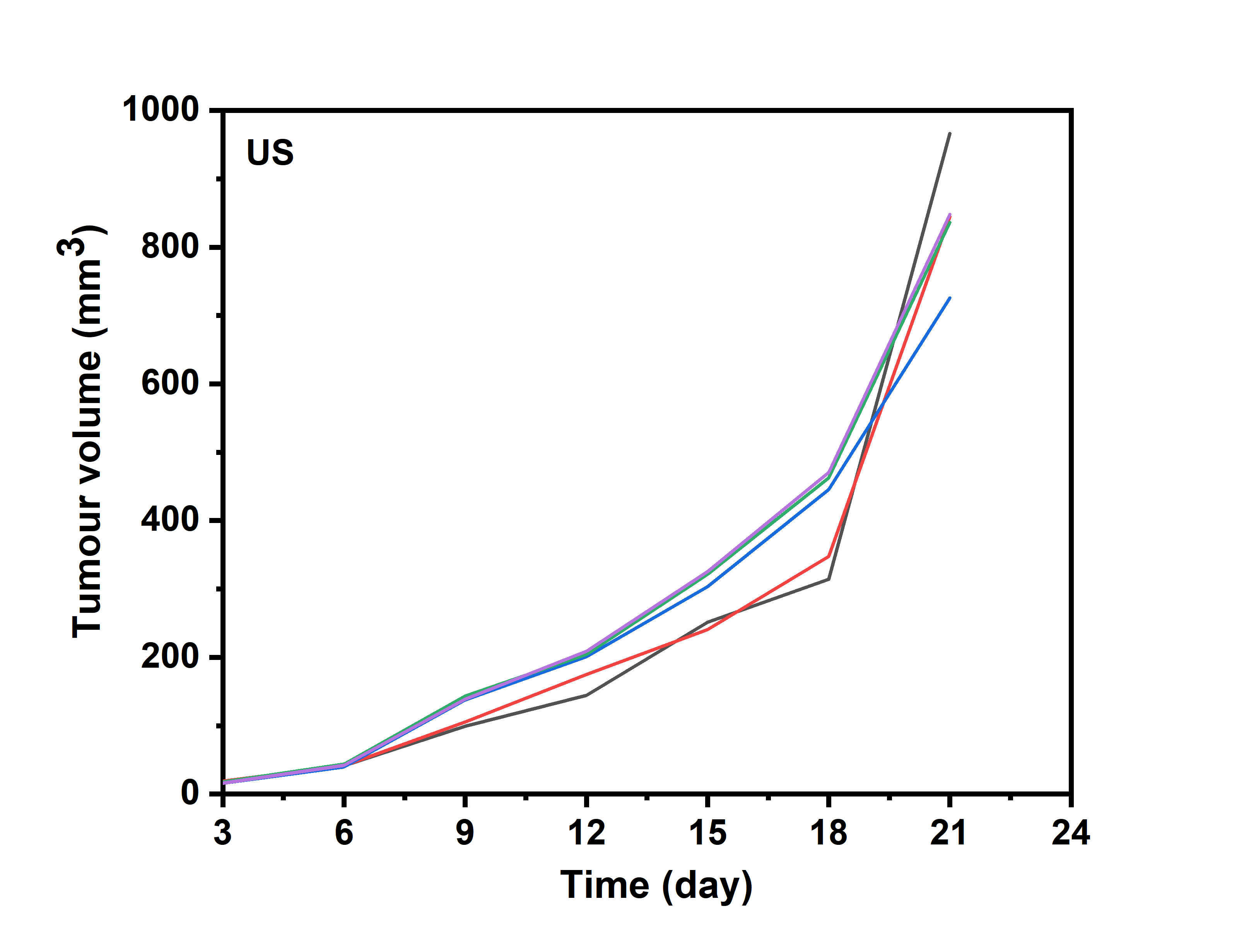


**Fig. S13.** The tumor volume of 4T1 tumor-bearing mice after treated with US.


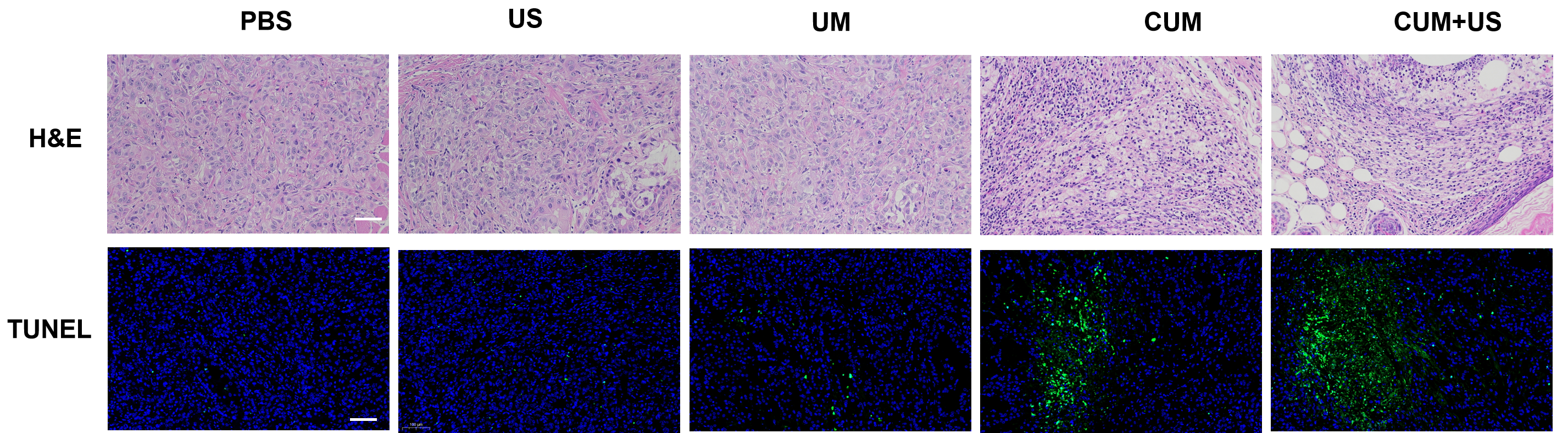


**Fig. S14.** H&E and TUNEL staining analysis of tumor tissues sections with different treatments. Scale bar: 100 μm.


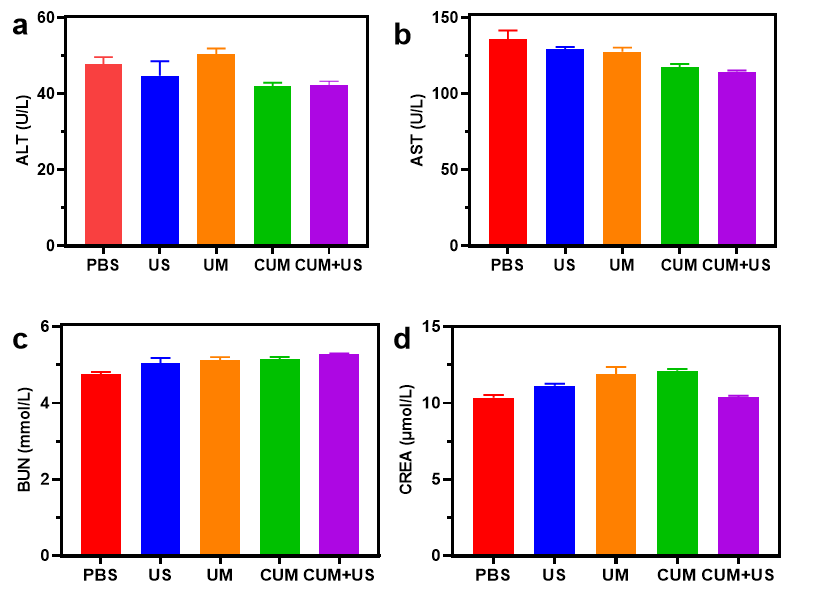


**Fig. S15.** The changes in ALT, AST, BUN and CREA levels of hematological indexes and biochemical data analysis in different treatment groups.


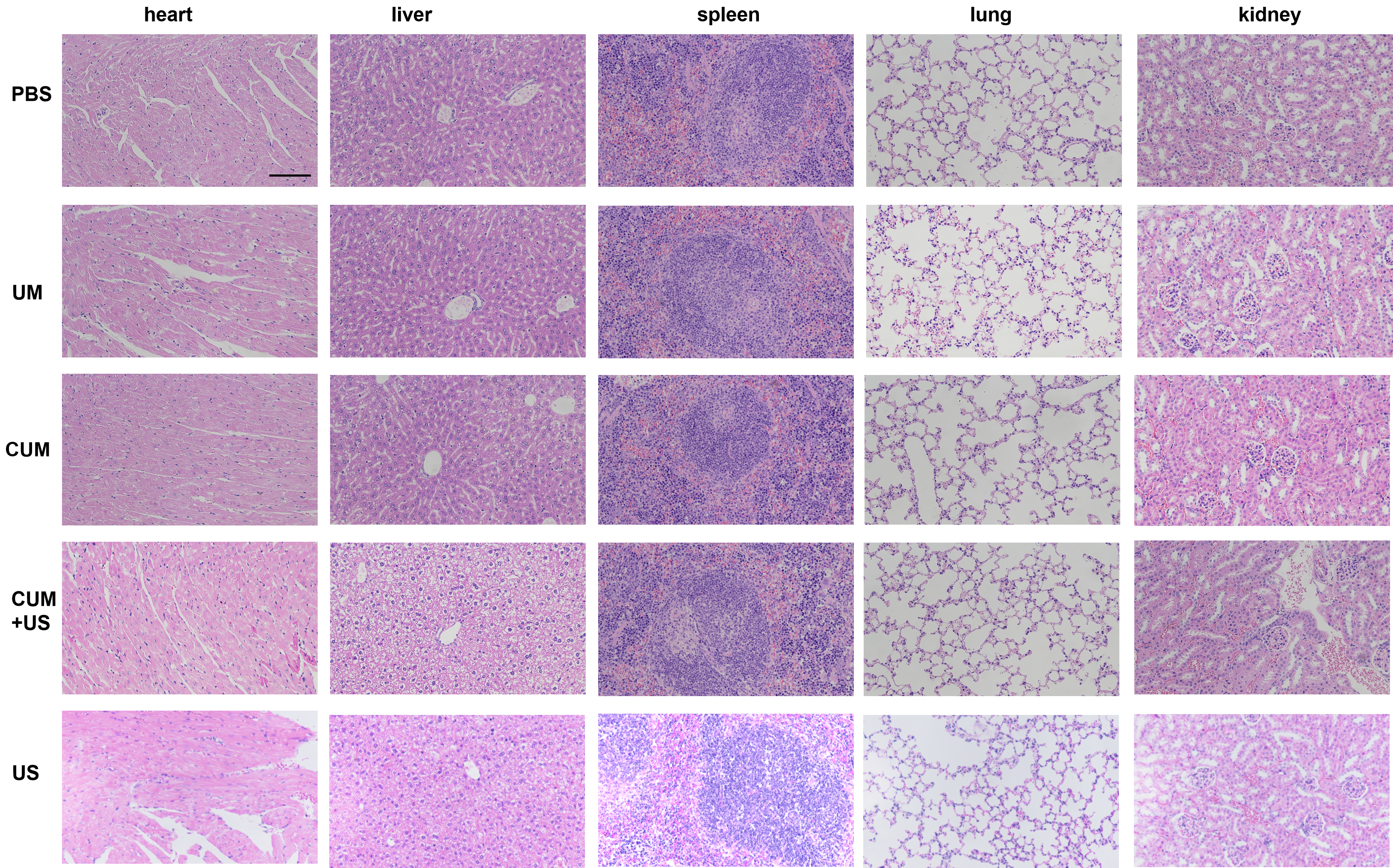


**Fig. S16.** H&E staining of PBS, US, UM, CUM and CUM+US within 14 days´ of treatment. Scale bar: 100 μm.


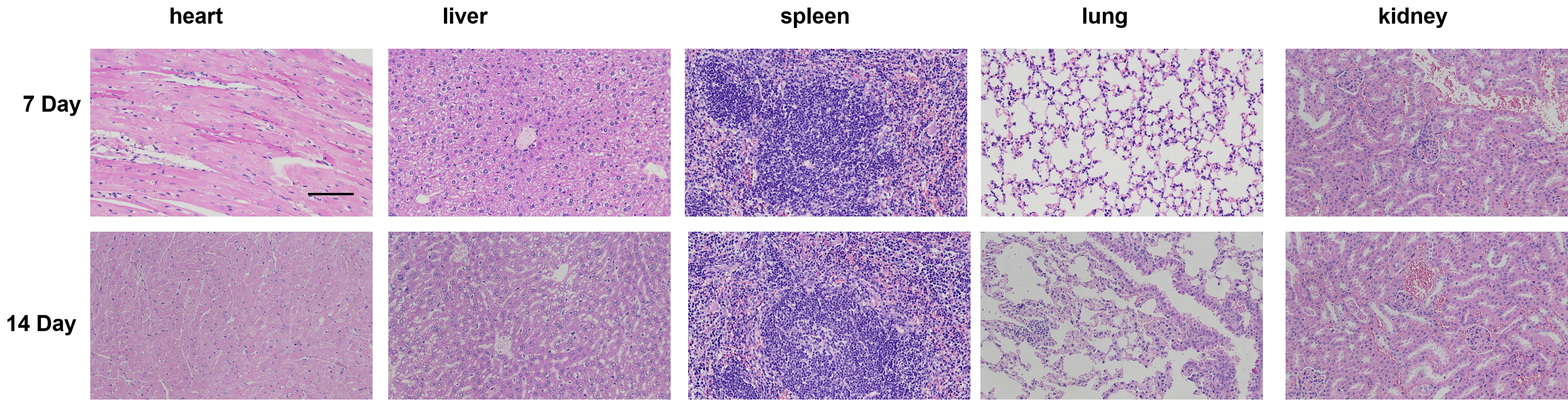


**Fig. S17.** The short-term toxicity assessment of CUM *in vivo*. Scale bars: 100 μm.


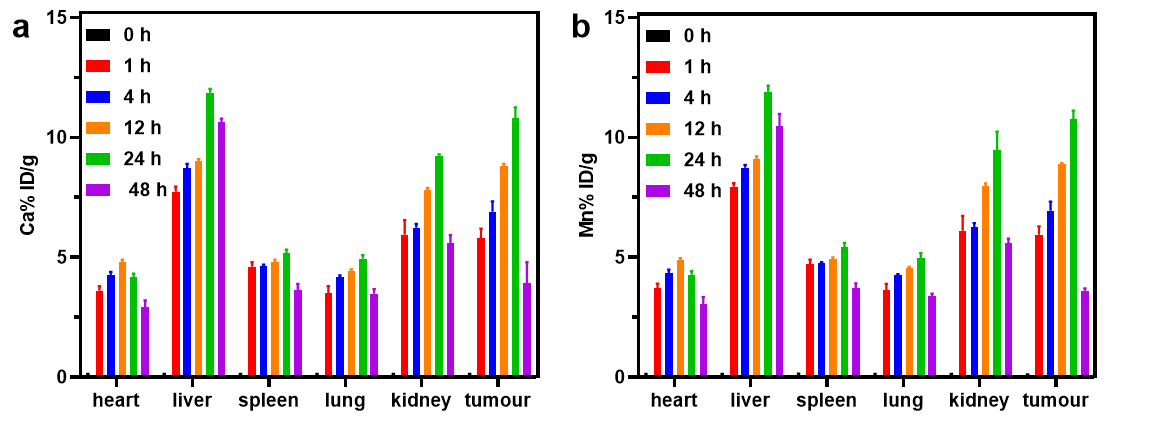


**Fig. S18.** (a,b) The biodistribution of Ca and Mn in main tissues at different times of post-injection of CUM (% injected dose (ID) of per gram of tissues).


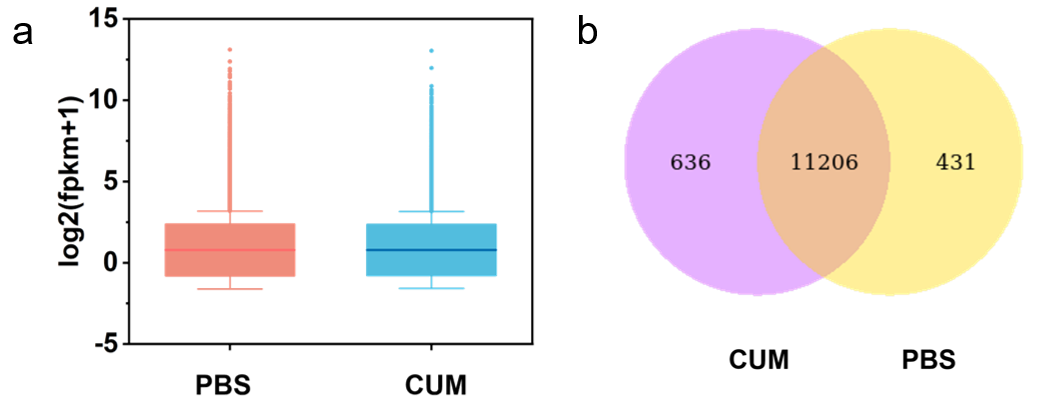


**a**

**b**

**Fig. S19.** (a,b) Genome sequencing analysis of the PBS versus CUM groups.


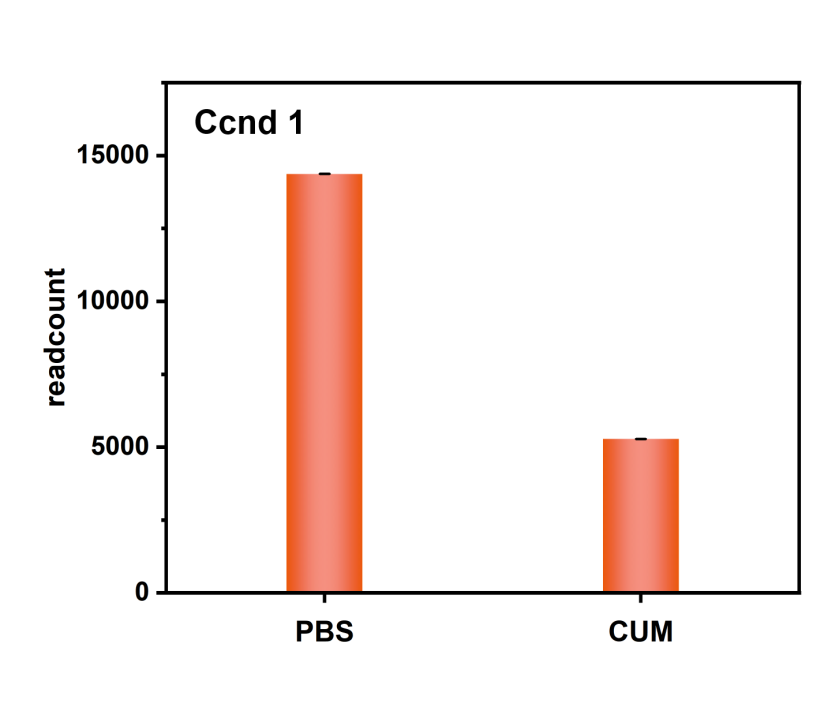


**Fig. S20.** Analysis of Ccnd 1 genome expression in the PBS and CUM groups.


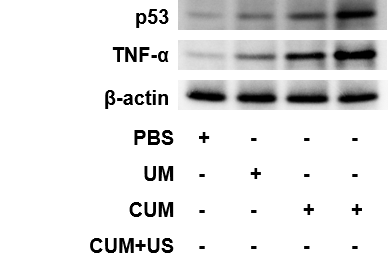


**Fig. S21.** Western blot analysis of pathway-related factors.

**Table S1**. Thermogravimetric analysis of UM and CUM.

| Group | Quality（mg) | TG (%) | CaCO_3_ (%) |
| --- | --- | --- | --- |
| UM | 3.73 | 48.63 | 6.37 |
| CUM | 3.50 | 38.88 |

With the masses of the UM group and CUM group used for TGA testing clearly known, the proportion of CaCO_3_ was calculated by comparing the difference in their mass losses, and the resulting calculated proportion of CaCO_3_ is 6.37%.

**Table S2**. EXAFS fitting parameters at the Mn K-edge for various samples (Ѕ_0_^2^=0.97 from MnO).


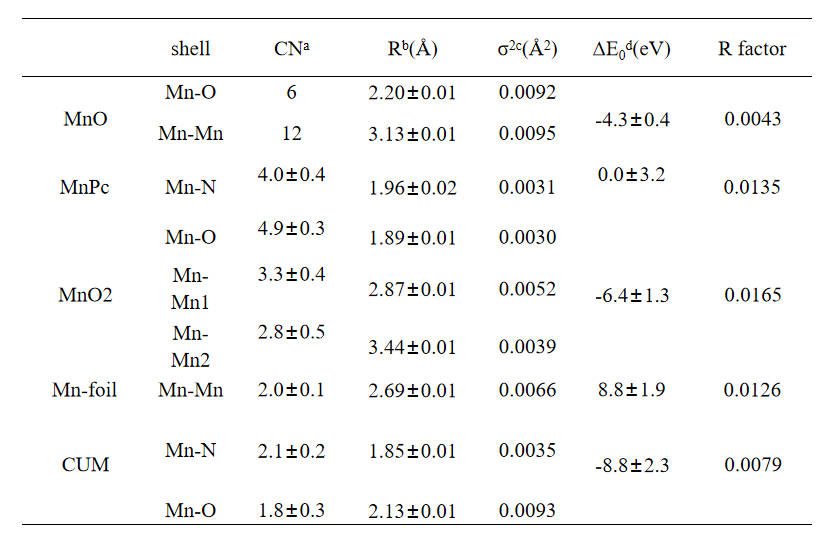

Supplement: Multimedia component 1 [file mmc1.doc]
